# Dynamic Interfacial Design in Adaptive Hybrid Materials Enables Reversible and Tunable Mechano-Optic Smart Responses

**DOI:** 10.1021/acsnano.5c22664

**Published:** 2026-06-25

**Authors:** Md Anisur Rahman, Vera Bocharova, Sungjin Kim, Jihye Choi, Bingrui Li, Sirui Ge, Monojoy Goswami, Xi Chelsea Chen, Catalin Gainaru, Alexei P. Sokolov, Tomonori Saito

**Affiliations:** † Chemical Sciences Division, 6146 Oak Ridge National Laboratory, Oak Ridge, Tennessee 37831, United States; ‡ Department of Materials Science and Engineering, University of Tennessee, Knoxville, Tennessee 37831, United States; § Department of Chemistry, 4292 University of Tennessee, Knoxville, Tennessee 37996, United States; ∥ Bredesen Center for Interdisciplinary Research and Graduate Education, University of Tennessee, Knoxville, Tennessee 37996, United States

**Keywords:** adaptive hybrid materials, vitrimers, reversible mechano-optical behavior, dynamic interface, programmable shape-memory behavior, self-assembly, microwrinkles

## Abstract

Next-generation polymeric materials are shifting toward adaptive and interactive behaviors of living systems; however, designing materials that can reversibly modulate optical properties under mechanical deformation while maintaining mechanical robustness remains a key challenge. Here, we report a mechanically robust vitrimer-based adaptive hybrid material (AHM) that exhibits a stretch-induced reversible transparency-to-opacity transition, enabled by the integration of dynamic interactions at the polymer–silica nanoparticle interface and controlled nanoparticle self-assembly. The AHM combines boronic ester–functionalized polystyrene-*b*-poly­(ethylene-*co*-butylene)-*b*-polystyrene (S-Bpin) with diol-functionalized silica nanoparticles (diol-SiNPs) to form a hybrid network hosting both dynamic boronic ester and hydrogen-bonding interactions. These reversible linkages facilitate controlled nanoparticle self-assembly and enable strain-induced nanoparticle alignment/aggregation. Upon stretching, SiNP-rich domains align and aggregate within the polymer matrix, while local modulus mismatch between stiff aggregated SiNP/borylated-styrene-rich regions and the softer elastomeric midblock induces surface microwrinkle formation. These internal aggregates and surface wrinkles cooperatively enhance light scattering, producing the opaque state under strain. Furthermore, the tailored AHM exhibits high toughness, thermomechanical stability, reprocessability, and programmable shape-memory behavior. This work presents a dynamic interfacial design strategy for mechanically robust, optically reconfigurable, and reusable soft materials for adaptive optics, smart windows, sensing, soft robotics, and circular smart-material platforms.

## Graphical abstract

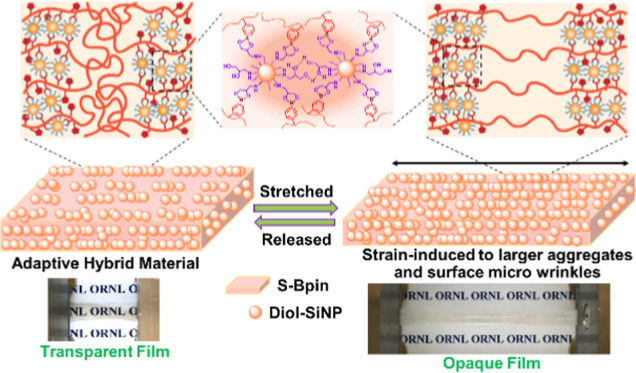

## Introduction

Next-generation polymeric materials are shifting toward adaptive and interactive soft materials, inspired by the dynamic behavior of living systems.^1–3^ These materials can sense, respond, and adapt to environmental changes by modulating their physicochemical properties, effectively mimicking the self-regulation and adaptability found in biological systems.^1,4–10^ They possess the ability to translate external stimuli, such as mechanical force, temperature, light, electromagnetic fields, or other environmental cues, into controllable changes in optical, mechanical, or structural properties. Such capabilities have enabled their growing use in flexible electronics,^11^ displays,^12^ smart windows,^13–16^ optical sensors,^17–20^ optical switches,^21^ soft robotics,^17,22^ reconfigurable coatings,^23^ actuators,^24,25^ biomedical devices,^2^ information storage or encryption technologies, and adaptive systems.^10,26–29^ As autonomous and intelligent soft matter technologies continue to expand, there is a growing need for smart adaptive materials that combine reversible function, programmability, mechanical robustness, and operation under practical conditions.

Traditional smart polymeric materials typically employ liquid crystals,^30^ suspended nano-/microparticles,^31^ photonic crystals,^32^ and phase-change materials,^23^ which can respond to external triggers such as light, temperature, or electric fields.^26^ Although effective in achieving tunable optical responses, there are existing challenges, including complex fabrication processes, relatively high energy consumption, and limited recyclability. Alternatively, mechanoresponsive hybrid systems, where mechanical deformation directly modulates optical or structural properties, have emerged as simpler, energy-efficient smart materials.^13,15,33,34^ Strategies employing microwrinkles on elastomers,^26,35^ carbon nanotubes,^36^ or graphene oxide films on elastomeric substrates and silica nanoparticle-embedded elastomers have demonstrated strain-induced transparency or color modulation.^37–40^ Despite these advances, most of these architectures rely on physically bonded nanoparticles or multilayered designs, which are prone to irreversible interfacial failure and delamination leading to optical fatigue during repeated mechanical cycling. The absence of dynamic interfacial chemistry restricts their durability, self-healing ability, reprocessability, and recyclability. Consequently, the creation of mechanically robust, healable, and recyclable smart adaptive materials that maintain both optical reversibility and structural integrity under deformation remains an important challenge.

Addressing this challenge requires new design paradigms that integrate dynamic polymer networks with precisely tailored polymer–nanoparticle interfaces. Achieving this goal demands new molecular design strategies that combine dynamic adaptability, interfacial stability, and controlled self-assembly, thereby enabling cooperative control of mechanical performance and functional responsiveness. Dynamic covalent networks, such as vitrimers, enable reversible bond exchange, facilitating stress relaxation, self-healing, and reprocessability without compromising cross-link density or structural integrity.^41–52^ When coupled with noncovalent interactions including hydrogen bonding (H-bonding) and supramolecular assembly, these systems could achieve synergistic control over energy dissipation, adaptability, and reversible reconfiguration. The incorporation of nanoparticles further extends this functionality: by tuning nanoparticle self-assembly and interfacial dynamics, the polymer matrix can maintain or reversibly modulate optical transparency.^13,53–56^ Such behavior parallels biological systems, where hierarchical organization and dynamic interfaces govern adaptive responses.^57^ Integrating dynamic covalent and noncovalent chemistry with nanoparticle-directed self-assembly thus offers a unique opportunity to bridge molecular-level reactive dynamics with nanoscale organization.^58–63^ However, the development of such materials with an adaptive polymer matrix linked to nanoparticles through reversible bonding that simultaneously combines mechanical adaptability with optically tunable functionality remains largely unexplored.

Here, we develop adaptive hybrid materials (AHMs) that integrate dynamic covalent and noncovalent interactions at a polymer–nanoparticle interface to achieve mechanically robust and tunable mechano-optical responses. The design combines boronic ester-functionalized polystyrene-*b*-poly­(ethylene-*co*-butylene)-*b*-polystyrene (S-Bpin) with diol-functionalized silica nanoparticles (diol-SiNPs). The diol-SiNPs form boronic ester linkages with the borylated styrene domains and can also associate through H-bonding among surface hydroxyl groups, promoting controlled nanoparticle self-assembly. The resulting AHMs combine reversible optical switching over repeated deformation, high toughness, thermomechanical stability, and reconfigurable shape-memory behavior. Structural analyses, surface imaging, and coarse-grained molecular dynamics (MD) simulations indicate that the room-temperature mechano-optical response arises from strain-induced nanoparticle alignment/aggregation and surface microwrinkling, whereas thermal activation enables boronic ester exchange, network relaxation, reprocessing, and shape reprogramming. This dynamic interfacial strategy provides a platform for optically adaptive, mechanically resilient, and reusable soft materials.

## Results and Discussion

### Design and Mechano-Optical Properties of AHMs

To validate our concept, we functionalized a triblock copolymer by incorporating dynamic covalent bond-forming groups. The styrene block of the SEBS triblock copolymer (118 kg mol^–1^, 30 mol % styrene) was selectively modified (∼90%) via aromatic C–H borylation to introduce dynamic boronic ester functionalities, forming S-Bpin, as reported in our earlier work.^1^ Boronic ester-functionalized triblock copolymer (S-Bpin) was employed, leveraging the ability of boronic ester groups to form dynamic covalent linkages with diol-SiNPs. The diol-SiNPs were synthesized through a two-step surface modification process from colloidal SiNPs (10–14 nm).^64^ First, amine and octyl groups (1:1) were introduced onto the nanoparticle surface using an amino silane and octyl silane coupling agents, followed by reaction with glycidol to introduce diol functionalities (Figure S1). Thermogravimetric analysis (TGA) confirmed the success of these modifications, as evidenced by distinct weight-loss events corresponding to each functionalization step (Figure S2a). Transmission electron microscopy (TEM) images of diol-SiNPs indicated that diol-SiNPs were interconnected/aggregated in tetrahydrofuran (THF) solution (Figure S2b). AHMs were prepared by dissolving S-Bpin and diol-SiNPs in THF to form a dispersed solution. The resulting mixture was dried under high vacuum to remove solvent. The partially dried AHM samples were subsequently dried at 120 °C under vacuum and then hot-pressed at 215 °C to prepare a film. During this thermal treatment, boronic ester groups of S-Bpin reacted with dihydroxyl groups on diol-SiNPs to form five-membered boronic ester linkages, creating a dynamic covalent cross-linked network (Figure 1a). Beyond these covalent bonds, the diol-SiNPs also present hydrogen-bond donors and acceptors (oxygen, nitrogen, and hydroxyl groups), enabling additional noncovalent hydrogen-bonding interactions that govern nanoparticle self-assembly (Figure 1b). The hot-pressed AHM film was mechanically robust and transparent (Figure 1c). Interestingly, the resulting AHM film exhibited two distinctive phenomena: (i) the formation of a metastable state after being stretched and released, which could be reverted to the ground state upon thermal stimulation [Figure 1d (left)], and (ii) a strain-induced reversible transition between transparent and opaque states [Figure 1d (right)]. To examine the influence of nanoparticle loading, AHMs containing 5, 10, and 20 wt % diol-SiNPs were fabricated (thickness ∼0.15–0.30 mm). All three compositions displayed strain-induced reversible opaqueness (Figure 1e and Table S1), demonstrating the optical tunability and mechanical robustness.

**Figure 1:**
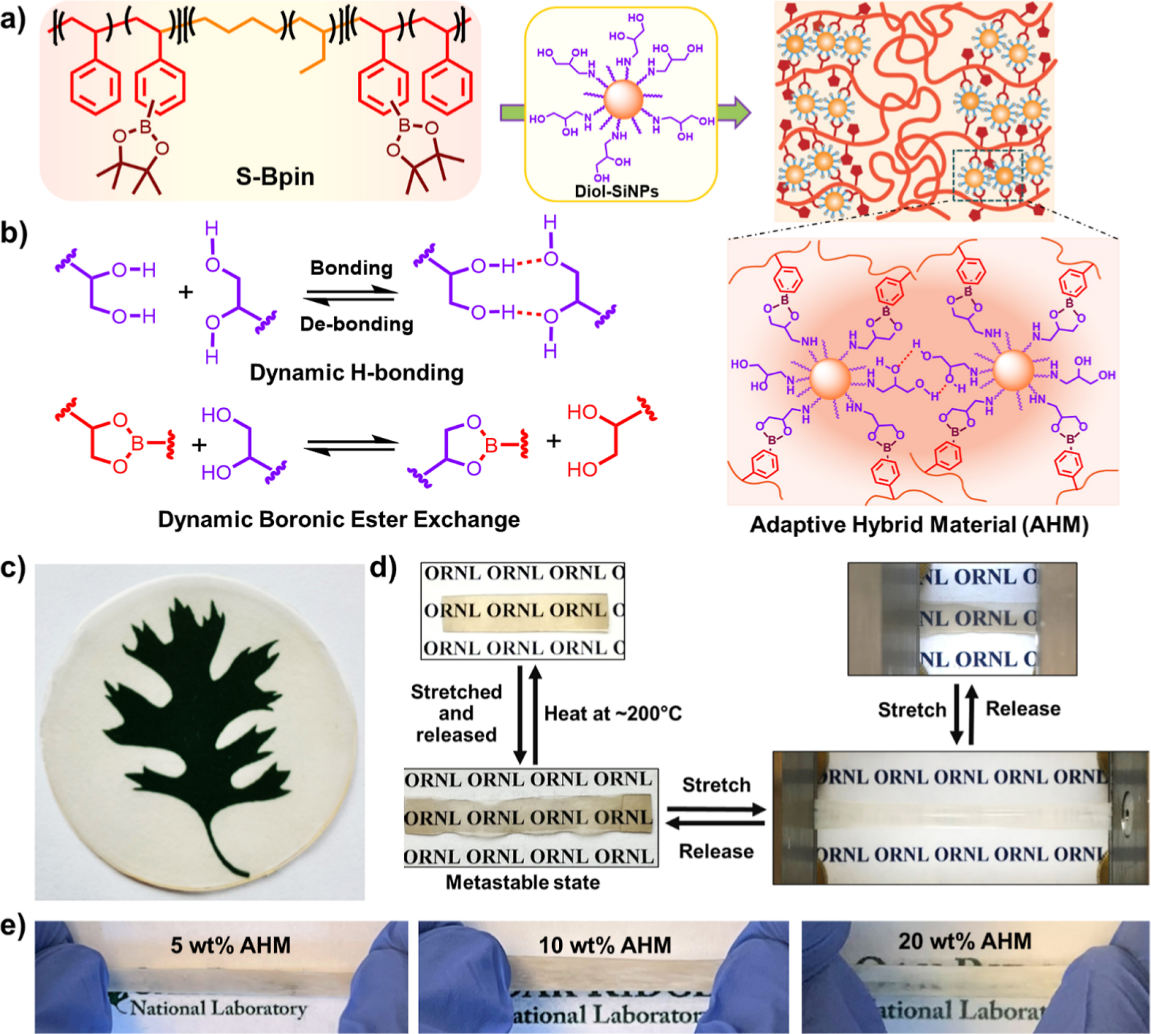
Design of AHMs and strain-induced reversible properties. (a) Fabrication of AHMs from boronic ester-functionalized commodity thermoplastic elastomer SEBS (S-Bpin) with diol-SiNPs. (b) Schematic of dynamic H-bonding interaction and boronic ester exchange at the diol-SiNP interface and S-Bpin-diol-SiNP interface. (c) Photographic image of 5 wt % diol-SiNP AHM film shows high transparency. (d) The image showing the strain-dependent transparency change and the metastable postdeformation state after stretching/release. (e) AHMs containing 5, 10, and 20 wt % diol-SiNPs exhibit strain-induced reversible opacity. Photo credit: Md Anisur Rahman, ORNL.

To illustrate the reversible opaqueness of AHMs, photographic images of the AHM film containing 5 wt % diol-SiNPs under varying tensile strains (0%, 10%, 20%, 50%, 100%, and 150%) are shown in Figure 2a. These AHM films exhibit a pronounced and progressive change in the optical behavior with increasing strain. At 0% strain, the film is transparent, allowing significant light transmission and clearly showing the letters behind the film. However, as the applied strain increases, the film becomes increasingly opaque. Notably, at 100% and 150% strain, the film appears almost completely nontransparent, demonstrating a strain-induced optical transition.

**Figure 2:**
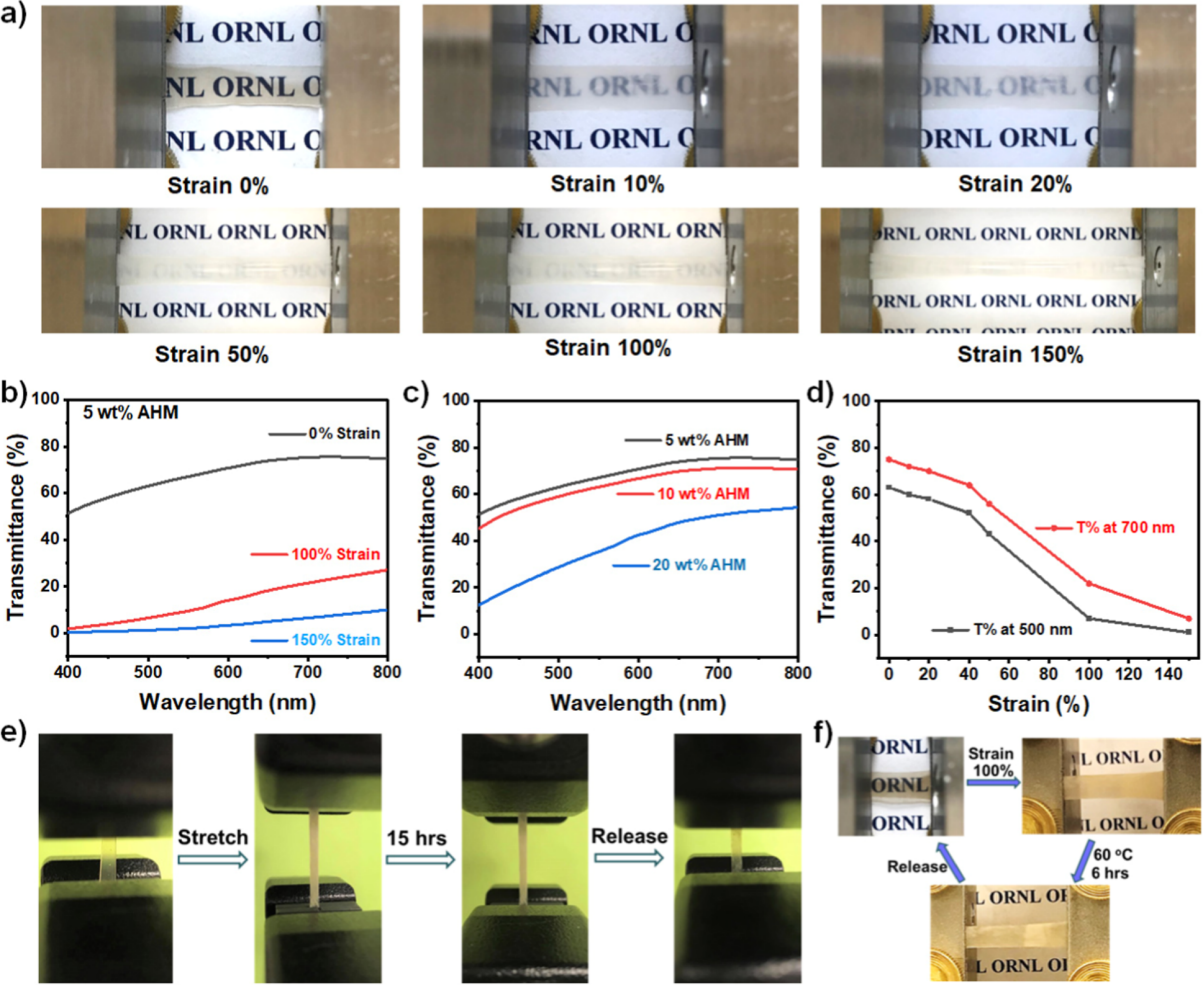
Strain-induced mechano-optical properties of AHMs. (a) Photographic images of AHM films at various strains demonstrating the strain-induced opaqueness/transparency change. (b) Optical transmittance spectra of 5 wt % diol-SiNPs containing AHM film at different strains. (c) Transmittance spectra of various diol-SiNP-loaded AHM and controlled samples at the initial state (0% strain). (d) Transmittance vs strain curves at wavelengths of 500 and 700 nm, respectively. (e) The photographic images demonstrate the opaqueness stability of AHM films over extended durations under high strain conditions. (f) Thermal stability of reversible transparency of AHM film over extended durations under high strain conditions. Photo credit: Md Anisur Rahman, ORNL.

To quantify the opaqueness response of the AHM films, UV–Vis–NIR spectroscopy was performed (Figure 2b). At 0% strain, the 5 wt % AHM films exhibited ∼75% transmittance in the visible region (400–700 nm). With increasing strain, light transmission decreased steadily: at 100% strain, the transmittance drops to 20–30%, and at 150% strain, it falls below 10%. This pronounced and measurable reduction in optical clarity confirms the strong correlation between mechanical deformation and optical response, underscoring the potential of these films for mechano-optical sensing, smart windows, or dynamic display applications.

We next examined the influence of diol-SiNPs loading on baseline transparency (0% strain) by varying the nanoparticle content from 5 to 20 wt %. As shown in Figure 2c, higher nanoparticle loading reduced the initial transparency. For example, AHM films with 10 and 20 wt % diol-SiNPs displayed ∼70% and ∼50% transmittance, respectively, compared with the ∼75% transmittance of the 5 wt % film. This reduction can be attributed to increased nanoparticle aggregation at higher loadings, which enhances light scattering. Transmittance vs strain curves (Figure 2d) of 5 wt % diol-SiNP AHM film at 500 and 700 nm wavelength showed that transmittance changes monotonously with strain. For example, the AHM film is still transparent at 20% strain, and the transparency reduces after 50% strain. The 5 wt % AHM film is almost completely opaque at 150% strain and showed transparency around 1% and 7% at 500 and 700 nm wavelengths, respectively. In contrast, films incorporating 10 wt % unfunctionalized SiNPs in SEBS or S-Bpin matrices appeared completely opaque (Table S1 and Figure S3), despite the ability of SiNPs to form boronic ester dynamic bonds with S-Bpin.^62^ This highlights the critical role of diol functionalization and dynamic covalent bonding at the polymer–nanoparticle interface in maintaining optical clarity. Notably, neat SEBS and S-Bpin films exhibited no detectable transparency changes under strain, confirming that diol-SiNP incorporation is essential for enabling strain-responsive optical modulation (Table S1). Furthermore, SEBS films containing 10 wt % diol-SiNPs retained partial transparency but were still less clear than the 10 wt % diol-SiNP S-Bpin films (Table S1). These findings establish that diol-SiNPs are essential for imparting strain-responsive optical functionality while preserving baseline transparency.

To evaluate the stability of strain-induced opacity, the films were subjected to 200% strain and held under constant strain for 15 h. No loss of opacity was observed during this period, and the films fully recovered their original transparency upon strain release (Figure 2e). To further probe temperature stability, a separate test was conducted by applying 100% strain and maintaining the stretched films at 60–65 °C for 6 h (Figure 2f). The films retained their opaque state under these conditions and again returned to their initial transparent state once the strain was removed.

To assess the durability of the reversible mechano-optical response through repeated cycling, the transmittance of the 5 wt % diol-SiNP AHM film at 700 nm was measured under 0% and 150% strain over 20 cycles, and the AHM consistently maintained its reversible mechano-optical response without significant loss in performance (Figure S4a). To further confirm the robustness of strain-induced optical switching, the 5 wt % AHM film was stretched and released more than 100 times manually and transmittance was measured at 0% and 150% strain. After 100 cycles, the film still exhibits approximately 70% transmittance at 0% strain and 20% transmittance at 150% strain at 700 nm (Figure S4b), indicating that the strain-dependent optical switching behavior is largely preserved over repeated deformation. These results demonstrate good cyclic mechano-optical stability under the tested conditions.

To elucidate the origin of strain-induced opacity, the morphology of AHM film was investigated by X-ray scattering. The wide-angle X-ray scattering (WAXS) and small-angle X-ray scattering (SAXS) profiles of 5, 10, and 20 wt % diol-SiNPs AHM films were compared to those of the parent polymer, S-Bpin (Figure 3a). The WAXS regions are nearly identical across all samples, with characteristic peaks indicating the amorphous nature of the materials. In contrast, the SAXS profiles changed significantly upon diol-SNP incorporation. For better resolution of peaks, the SAXS data are presented in the form of a Kratky plot (Figure 3b). S-Bpin showed a primary peak at *q*_1_ ≈ 0.017 Å^–1^ and a weak second-order peak at *q*_2_ ≈ 2*q*_1_, corresponding to a domain spacing of ∼35 nm, which is consistent with weakly ordered lamellar microphase separation reported for related SEBS-based systems.^65^ Upon incorporation of diol-SiNPs, a weaker scattering feature emerged corresponding to a smaller domain size of ∼15 nm at *q* = 0.04 A^–1^, which corresponds to the nanoparticle size. The dihydroxyl groups in diol-SiNPs react with boronic ester groups in the S-Bpin triblock copolymer, promoting preferential localization of the nanoparticles within the borylated styrene domains. Alternatively, due to the smaller size of diol-SiNPs and hydrophilicity, the nanoparticles may also reside in the more hydrophilic regions of the polymer matrix. They contribute to low-*q* scattering (0.003 A^–1^ to 0.03 A^–1^) through center-to-center correlations (*d*_NP_) and can enhance scattering from the phase-separated polymer (*d*_polymer_) by increasing the scattering length density contrast. These contributions cannot be easily separated from SAXS alone. To further probe the structural evolution under stretch, in situ SAXS was performed on the 5 wt % diol-SiNP AHM at strains of 10, 20, 40, 50, 100, 150, and 200% (Figure 3c and a corresponding Kratky plot in Figure 3d). Increasing strain led to enhanced low-q scattering, suggesting the formation of larger SiNP-rich aggregates during stretching. This behavior implies strain-induced reorganization of the nanoparticle–polymer network, resulting in stronger aggregation of SiNPs. The corresponding 2D SAXS patterns show a transition from an isotropic circular halo (Figure 3e) in the unstrained state to an anisotropic “butterfly” pattern at high strain (Figure 3f), reflecting nanoparticle alignment and nanoparticle aggregation along the strain direction. Although very large aggregates exceed the SAXS detection window, the broadening and intensification of the low-*q* signal support deformation-driven nanoparticle reorganization.

**Figure 3:**
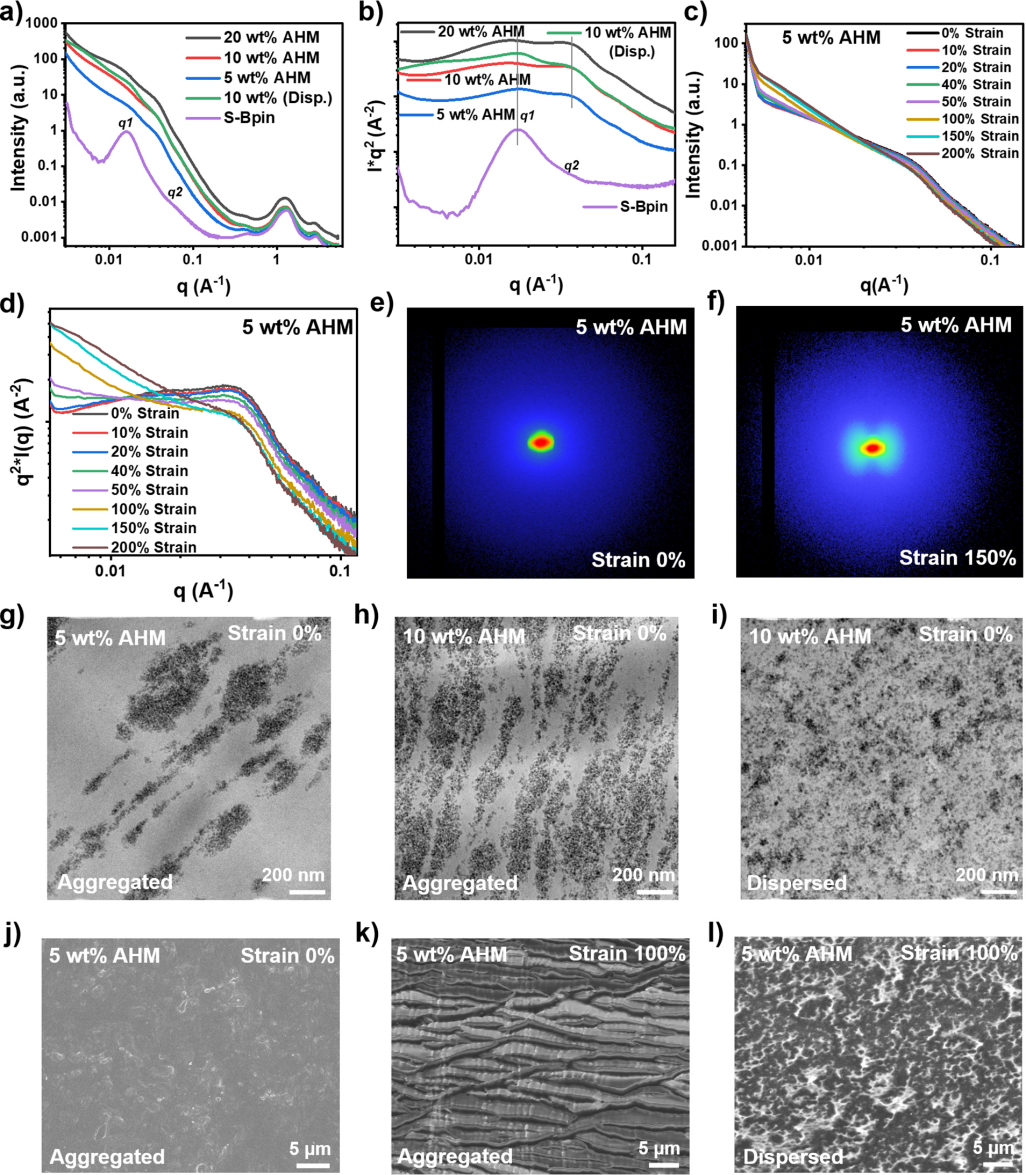
Morphology of SiNPs and strain-induced surface wrinkles in AHMs. (a) SAXS and WAXS of AHM films of various SiNP loading. (b) Kratky plot of SAXS data shown in (a). (c) SAXS patterns of 5 wt % AHM film under different strains. (d) Kratky plot of SAXS data for 5 wt % AHM film under different strains. (e) 2D SAXS pattern of 5 wt % AHM film at 0% strain. (f) 2D SAXS pattern of 5 wt % AHM film at 150% strain. (g) TEM images of 5 wt % AHM film (aggregated) at 0% strain. (h) TEM images of 10 wt % AHM film (aggregated) at 0% strain. (i) TEM images of AHM film with 10 wt % AHM (dispersed) at 0% strain. (j) Scanning electron microscopy (SEM) image of 5 wt % AHM film (aggregated) at 0% strain. (k) SEM image of 5 wt % AHM film (aggregated) at 100% strain formed microwrinkles. (l) SEM image of the well-dispersed 5 wt % AHM after 100% strain, showing no oriented wrinkle formation.

To further elucidate the real-space visualization of morphological transformations, TEM was conducted on AHM film with 5 wt % (Figure 3g) and 10 wt % (Figure 3h) diol–SiNPs at 0% strain. The TEM images revealed heterogeneous morphologies where diol-SiNPs form both aggregated clusters and aligned string-like assemblies (Figure S5). This coexistence of clustered and anisotropic structures indicates partial nanoparticle association within the borylated styrene domain. To verify the role of nanoparticle aggregation, a control AHM containing well-dispersed 10 wt % diol–SiNPs was synthesized (Figure S6a). The TEM image of this sample shows a homogeneous dispersion of nanoparticles without noticeable large clustering (Figure 3i). The SAXS spectra of 10 wt % diol-SiNPs (dispersed) are characterized by the presence of two peaks in the low-q region (Figure 3b): one coinciding with the scattering from the polymer at *q* = 0.017 Å^–1^ (*d* = 35 nm), and another at *q* = 0.005 Å^–1^ (*d* = 120 nm). Based on the TEM image, diol-SiNPs are homogeneously distributed at distances less than 200 nm. This indicates that center-to-center scattering from nanoparticles would also contribute to low-*q* X-ray peaks. The presence of individually dispersed particles and dispersion of smaller aggregates are also supported by the TEM image (Figure 3i). Notably, this well-dispersed film did not exhibit strain-induced opacity (Figure S6b), confirming that controlled nanoparticle aggregation/string-like organization is essential for strong light scattering under strain.

Surface morphology was also examined to determine whether surface instabilities contribute to the optical response. Optical microscopic images of the 5 wt % diol–SiNP AHM under different strains were taken (Figure S7) and revealed no macroscopic cracks, damage, or large-scale surface wrinkles, even after 150% strain. However, because optical microscopy at this length scale may not resolve nanoscale surface morphologies, SEM of the 5 wt % diol–SiNP AHM (Figures 3j,k) was further performed to examine the strained surface morphology with higher resolution. The unstrained film (Figure 3j) displays a smooth surface, whereas at 50% and 100% strain (Figure S8), oriented wrinkle-like features become apparent (Figure 3k). The wrinkle formation is attributed to the local modulus mismatch between stiff diol-SiNP/borylated-styrene-rich domains and the softer poly­(ethylene-*co*-butylene) midblock. Dynamic boronic ester linkages between diol–SiNPs and borylated styrene domains reinforce the SiNP-rich regions, while the soft midblock maintains a lower modulus. Under tensile deformation, this mechanical heterogeneity promotes oriented microwrinkle formation, which provides additional light-scattering interfaces and amplifies the opacity generated by internal nanoparticle aggregation. In contrast, 5 wt % AHM with well-dispersed nanoparticles did not show oriented microwrinkles after 100% strain (Figure 3l) and did not exhibit strain-induced opacity. These results indicate that surface wrinkling contributes to the optical switching, but only when SiNPs are preorganized into aggregated/string-like domains.

### Thermal, Mechanical, and Dynamic Properties of AHMs

To evaluate the mechanical robustness of the AHM films, uniaxial tensile tests were conducted on films with varying diol-SiNP loadings. The stress–strain curves of all films displayed characteristic thermoplastic elastomer behavior, including high initial stiffness, necking, a defined yield point, followed by strain hardening and large elongation at break (Figure 4a). The tensile strength increased with diol-SiNP content up to 10 wt %, reaching ∼39 MPa, but decreased at higher loading (33 MPa at 20 wt %; Figure S9 and Table S2). For comparison, the 5 wt % film exhibited ∼37 MPa tensile strength. Interestingly, the 5 wt % diol-SiNP AHM showed exceptional toughness (∼83 MJ m^–3^), significantly higher than the other formulations (Figure 4b, Table S2). These results suggest that moderate nanoparticle incorporation reinforces the polymer network, while higher loading promotes aggregation, which affects mechanical performance. To further evaluate the durability and elasticity of the 5 wt % AHM film, cyclic tensile tests were conducted at 100% and 150% strain for five consecutive cycles (Figures S10 and 4c). The resulting stress–strain curves revealed that the AHM film exhibited noticeable hysteresis during the first cycle, followed by good cyclic stability with minimal hysteresis from the second cycle onward. This behavior indicates efficient energy recovery and excellent elastic resilience of the AHM network. The larger hysteresis observed in the first cycle is attributed to nanoparticle/aggregate realignment and polymer network rearrangement, leading to the formation of a metastable state after unloading. Importantly, this hysteresis was fully recoverable upon mild heating (as shown in Figure 1d). After the initial cycle, the AHM films endured repeated large deformations without significant loss of mechanical integrity, underscoring the contribution of dynamic interactions and robust interfacial bonding to the film’s reversible deformation behavior.

**Figure 4:**
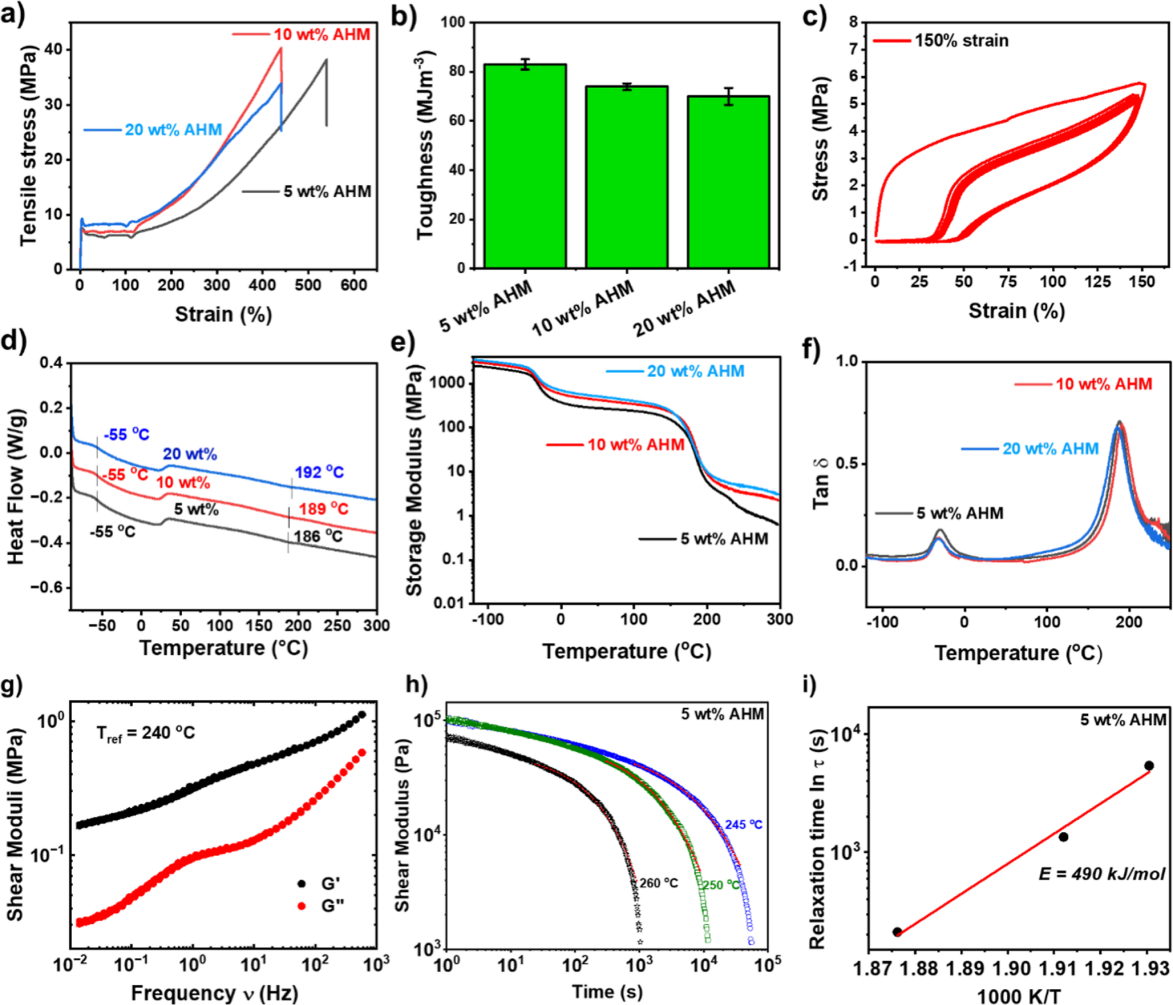
Mechanical and thermal properties of AHM film. (a) Representative tensile stress–strain curves of AHMs. (b) The toughness of different diol-SiNPs containing AHMs. (c) Hysteresis test for AHM film. (d) *T*_g_ values were determined by differential scanning calorimetry (DSC). (e) The shear storage modulus of AHMs as a function of temperature measured by dynamic mechanical analysis (DMA). (f) The tan delta curves from DMA of AHMs clearly indicate two different glass transition temperatures. (g) Master curves of real and imaginary parts of complex shear modulus for 5 wt % diol-SiNP AHM, constructed using as reference temperature 240 °C. (h) Time-domain shear relaxation curves for the 5 wt % AHM. Dashed lines are fits using Kohlrausch–Williams–Watts functions. (i) Characteristic time of terminal flow extracted for the 5 wt % AHM. The solid line is an interpolation using the Arrhenius equation.

DSC was employed to determine the glass transition temperatures (*T*_g_) of all AHM films. Each sample exhibited two distinct *T*_g_ values: one near −55 °C, corresponding to the soft ethylene-butylene middle block of S-Bpin, and another between 180 and 195 °C, associated with the boronic ester–functionalized styrene block (Figure 4d). As expected from two distinct *T*_g_ from DSC, DMA further revealed that all AHM films possessed a significantly broader service temperature window at −55–∼192 °C (Figure 4e), a desirable attribute for applications requiring wide operational temperature ranges. The storage modulus increased with higher SiNPs loading and displayed a secondary plateau at temperatures above the second *T*_g_. This behavior indicates the formation of robust dynamic covalent cross-links between the SiNPs and the polymer matrix. The tan δ plots (Figure 4f) also confirmed the presence of two distinct *T*_*g*_s: one at approximately −30 °C and another between 186 and 192 °C. The high-temperature *T*_*g*_ of the borylated polystyrene block showed a slight upward shift with increasing diol-SiNP loading up to 10 wt %, likely due to enhanced cross-link density that restricts chain mobility. For example, the *T*_*g*_ of the borylated polystyrene block in the 10 wt % diol-SiNP AHM was 192 °C, compared to ∼188 °C for the 5 wt % AHM formulation. These findings demonstrate that increasing nanoparticle content not only improves the stiffness of the AHMs but also extends their usable temperature window. The interfacial dynamic bonding between the SiNPs and polymer matrix plays a key role in stabilizing the material’s structure across a wider temperature range, thereby enhancing its mechanical robustness and thermal adaptability.

To gain additional information on the dynamic characteristics of the AHMs, oscillatory shear rheology was employed to access the viscoelastic response of 5 wt % diol-SiNP AHM film. The corresponding master curves of real and imaginary parts of the complex shear modulus, G* = *G*′ + *iG*″ are presented in Figure 4g. These master curves were constructed by horizontally shifting spectra recorded at different temperatures between 220 and 260 °C on top of the one corresponding to 240 °C. In this temperature range, the two moduli exhibit a decrease starting from high frequencies, followed by a plateauing in the intermediate dynamic range, and another softening for frequencies below 1 Hz. Interestingly, the corresponding shear tan delta (= *G*″/*G*′) master curve in Figure 4h exhibits a maximum, revealing that the slow dynamics is in fact dominated by an underlying relaxation process. Considering the temperature range in which it emerges and its relatively weak contribution to *G*′ (in the sub-MPa range, see Figure 4g), this shear relaxation feature corresponds to the small decay observed for storage modulus above 200 °C (Figure 4e). The corresponding characteristic times extracted from the position of this maximum are plotted as a function of inverse temperature in Figure S11a. These data can be interpolated relatively well with an Arrhenius law [τ=τ_0_·exp­(*E*_*a*_/*RT*), with *R* being the gas constant] with a prefactor τ_*0*_ = 10^–15.7^s and an activation energy *E*_*a*_ = 320 kJ/mol (Figure S11b), which is attributed to softening of the borylated polystyrene domains rather than terminal flow.

To evaluate the terminal flow characteristics of the 5 wt % AHM film, shear stress-relaxation measurements were performed on the 5 wt % diol–SiNP AHM at elevated temperatures under a constant strain of 1%. The results, illustrated in Figure 4h, show that the shear modulus decreases over long times in a double logarithmic plot, eventually dropping to less than 1% of its initial value. This significant decay confirms the existence of terminal flow, indicating that these materials are processable by flow. To quantify the relaxation dynamics, the data were fitted using Kohlrausch functions, F­(*t*)∝exp­[-(*t*/τ_0_)^β^], where τ_0_ represents the characteristic relaxation time, and β is the stretching parameter. Using these two fitting parameters, the mean relaxation times τ_*F*_ were estimated as τ = τ_0_/β×Γ­(1/β), with Γ representing the gamma function. The corresponding results are included in Figure 4i, which reveal an activation energy of 490 kJ/mol, which aligns with values previously observed for boronic ester covalent bond rearrangements.^59,62,66^ The higher activation energy reflects the combined contributions of segmental relaxation, dynamic boronic ester bond exchange, and polymer chain disengagement from nanoparticle-rich aggregates. Similar stress-relaxation measurements were attempted for the 10 and 20 wt % diol–SiNP AHM films, and their corresponding relaxation responses are shown in Figure S12a,b, respectively. These higher-loading samples exhibited much slower relaxation. Complete terminal decay would require prolonged measurements over several days at elevated temperatures, where thermal degradation becomes significant. Therefore, reliable activation energies could not be extracted for the 10 and 20 wt % AHMs. Nevertheless, the partial responses obtained after 1 day of probing, as shown in Figure S12, indicate that flow does occur in principle, further supporting the processability of these materials. The slower relaxation at higher nanoparticle loading is consistent with an increased density of polymer–nanoparticle interfacial interactions, which delays network rearrangement and terminal flow. H-bonding among hydroxyl-functionalized SiNPs likely contributes to nanoparticle association and reorganization, although direct spectroscopic quantification of hydrogen-bond exchange under these conditions was not performed.

The reversible boronic ester exchange between polymer-bound boronic esters and SiNP surface diols, together with H-bonding among nanoparticles, forms a dynamic and reprocessable network (Figure 5a). This reversible chemistry enables multiple reprocessing cycles without significant performance loss, as thermal activation facilitates nanoparticle realignment and network rearrangement. The reprocessed films also maintained strain-induced reversible opaqueness (Figure 5b), exhibiting ∼60% transmittance at 0% strain and becoming nearly opaque at 150% strain (Figure 5c). Tensile stress–strain analysis further revealed that the reprocessed samples retained mechanical integrity after multiple cycles; notably, the 5 wt % diol-SiNP AHM maintained comparable mechanical behavior after two reprocessing events, confirming the robustness of the reversible interfacial bonding and the absence of significant chain scission or network collapse (Figure 5d). Fourier transform infrared (FTIR) analysis confirmed that the reprocessed films retained their chemical integrity, showing spectra nearly identical to the pristine sample (Figure 5e). These observations demonstrate that dynamic bonding between diol–SiNPs and S–Bpin polymers enables network reorganization during thermal reprocessing while preserving optical reversibility.

**Figure 5:**
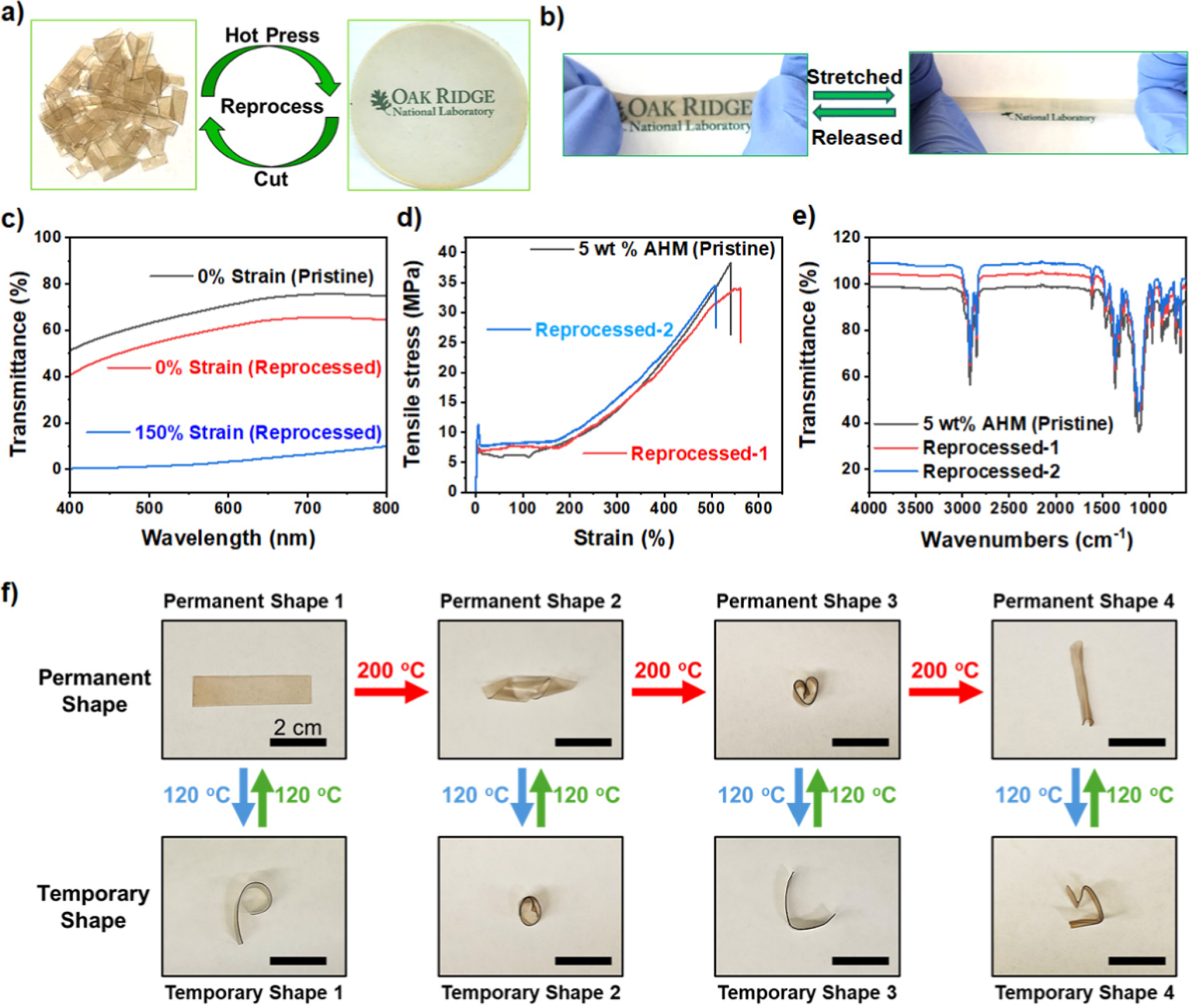
Adaptive behavior of AHM. (a) Reprocessability of 5 wt % diol-SiNP AHM under 200 °C. (b) Reprocessed film exhibited the strain-induced opaqueness similar to the pristine sample. The photograph of the reprocessed sample shows the excellent reversible transparency. (c) Transmittance spectra of the reprocessed diol-SiNP-loaded dynamic composite film at various strains and compared with the pristine sample. (d) Tensile stress–strain curves of the reprocessed AHM film. (e) FTIR of reprocessed 5 wt % diol-SiNP AHM. (f) Reprogrammable shape-memory behavior of AHM. Photo credit: Md Anisur Rahman, ORNL.

### Shape-Memory Behavior of AHM

The AHM exhibits excellent reprogrammable shape-memory behavior, as illustrated in Figure 5f. This behavior arises from the combined effects of elastomeric recovery, nanoparticle alignment, and thermally activated dynamic interfacial exchange. Interestingly, the AHM transitions into a metastable state after the initial stretch is released. The metastable state observed after stretching and release is interpreted as a kinetically trapped postdeformation morphology rather than a new equilibrium state. During stretching, the soft poly­(ethylene-*co*-butylene) midblock stores elastic energy and transfers stress to the SiNP-rich borylated styrene domains, promoting nanoparticle alignment/aggregation and wrinkle formation. Upon strain release, the entropic elasticity of the midblock provides the driving force for macroscopic recovery. However, complete recovery of the initial nanoscale morphology can be delayed because the reorganized SiNP-rich domains are stabilized by interparticle H-bonding, confinement within the stiffer borylated styrene domains, and polymer–nanoparticle interfacial interactions. Thermal stimulation (heating at ∼200 °C) increases polymer mobility and activates interfacial relaxation, including boronic ester exchange at elevated temperature, allowing the system to overcome these kinetic barriers and return toward the equilibrated ground state (Figure S13a). Thus, the metastable behavior arises from a balance between entropy-driven elastic recovery and enthalpically stabilized, kinetically trapped nanoparticle/interfacial structures. The AHM exhibits two distinct *T*_g_ between −55 °C and ∼190 °C, enabling multistage shape manipulation. At temperatures within this range, a temporary shape can be fixed, whereas heating above 200 °C activates dynamic interactions between diol–SiNPs and borylated styrene units, facilitating network rearrangement and resulting in programmable, reconfigurable shape-memory behavior. In contrast to conventional shape-memory polymers, which possess a single permanent shape determined by their cross-linked network, the AHM’s reconfigurable vitrimer network enables plasticity-driven permanent shape change upon thermal activation. For example, an initial rectangular film (permanent shape 1) was heated to 120 °C (*T* > *T*_g_) for 5 min, deformed into the letter “P” (temporary shape 1), and cooled to room temperature to fix the shape. Upon reheating to 120 °C, the original rectangular form was fully recovered. To reprogram the permanent shape, the same film was twisted at 200 °C (*T* > *T*_r_) and held for 1 min, inducing network rearrangement that established a new equilibrated shape (permanent shape 2) upon cooling. Using this protocol, multiple permanent shapes, including heart (shape 3) and bent (shape 4) forms, were fabricated. Each permanent configuration could be temporarily fixed into shapes such as “P,” “O,” “L,” and “Y”, demonstrating a one-way shape-memory effect. This switching behavior originates from the dynamic interactions and network rearrangement, which undergo bond exchange at elevated temperatures, facilitating stress relaxation and shape recovery. The 5 wt % AHM exhibited a shape fixity ratio of 85.2% and a shape recovery ratio of 56.1% (Figure S13b). The reconfigured shape showed a shape fixity ratio of 88.4%, confirming effective programming of a new permanent shape. The ability to repeatedly program and recover complex shapes underscores the material’s potential for reconfigurable smart structures, adaptive devices, and soft robotic applications.

### MD Simulations

To obtain a fundamental understanding of the effect of interactions between borylated styrene and diol-SiNPs on the agglomeration of NPs under strain, coarse-grained (CG) MD simulations were carried out. The focus of the MD simulations was to investigate the physics of agglomeration under strain as a function of interaction strengths between the polymer and NPs. Therefore, a polymer model was used to simulate polymer-NP behavior under strain without constraining the simulations with details of diol grafting on SiNPs and polydispersity. To reduce the complexities of simulating the system, we developed a simpler simulation model following the Kremer–Grest (KG) bead–spring model,^67^ in which monomers are connected by finite extensible nonlinear elastic (FENE) bonds. The styrene groups are modeled by fixed rigid side beads connected by FENE bonds, and the SiNPs are modeled as spherical particles consisting of beads (Figure S14). The simulation system replaces the complexity in the computation of diol-grafted SiNPs with enhanced interaction parameters between the polymer and SiNPs representing the effect of diol grafting. The polymer–SiNP interactions are governed by van der Waals forces represented by a tunable Lennard-Jones (LJ) potential. The NP–polymer side bead interactions are stronger, representing strong interactions of rigid styrene beads with diol-SiNP, while the polymer backbone–NP interactions are kept weak. The simulations are carried out in a box with periodic boundary conditions in the *x*- and *y*-axes, while the *z*-axis is fixed. The whole system is equilibrated for 5 million LJ time steps in the *NVT* ensemble, followed by 4 million LJ time steps in NPT. The system is then further equilibrated for 35 million LJ time steps. Strain is applied for 5 million LJ time steps by introducing a weak constant velocity along the *y*-axis that moves all the particles along the *y*-axis. From the fully strained system, the strain is released by the same method, that is, by reducing the velocity at the same rate. While the simulations are simplified to achieve computational efficiency, CG MD simulations of the KG model have proven to have a record of providing an excellent explanation of the fundamental physics of the polymer-NP composites.^68^ The details of the simulation can be found in the Supporting Information.

Before applying strain, fully equilibrated NPs are seen in Figure 6a (for clarity, the polymer chains are not shown; the full system is shown in Figure S15a). Once strained using constant velocity *z*-axis stretching, the NPs move away, developing clusters and string-like structures as observed in TEM images (Figures 6b and S15a2). Once the strain is released (contracted), the AHM system returns to its original state (Figures 6c and S15a3). The radial distribution function (RDF) (blue line, Figure S15b) between the side beads near 5s (where s is the bead diameter) from the SiNP and nanoparticle shows a layered, highly agglomerated (strong peak) NP structure surrounded by side beads when strain is applied. Once the strain is released, the RDF peaks (red line, Figure S15b) become weaker, representing weaker agglomeration between NPs and side beads while maintaining the layered structure. This corroborates the experimental finding of strain-induced NP agglomeration in stringlike smaller structures within the styrene domains. The reversible mechanoresponse is confirmed by observing the potential energy (PE) response with strain during stretching and contraction. Strain is applied and released in two consecutive stages with no change in PE during stretching and contraction, i.e., no hysteresis of the PE curve and hence no energy loss, representing a fully reversible mechano-response system (Figure S15c). The density distribution (Figure S15d) of those side beads residing within 5s from the NP exhibits side beads clustering around the NP peaks in both the strained (blue) and strain-released states. Because the CG model does not include explicit dynamic covalent chemistry, these simulations should be interpreted as evidence for reversible nanoparticle clustering and alignment physics, rather than direct proof of bond exchange.

**Figure 6:**
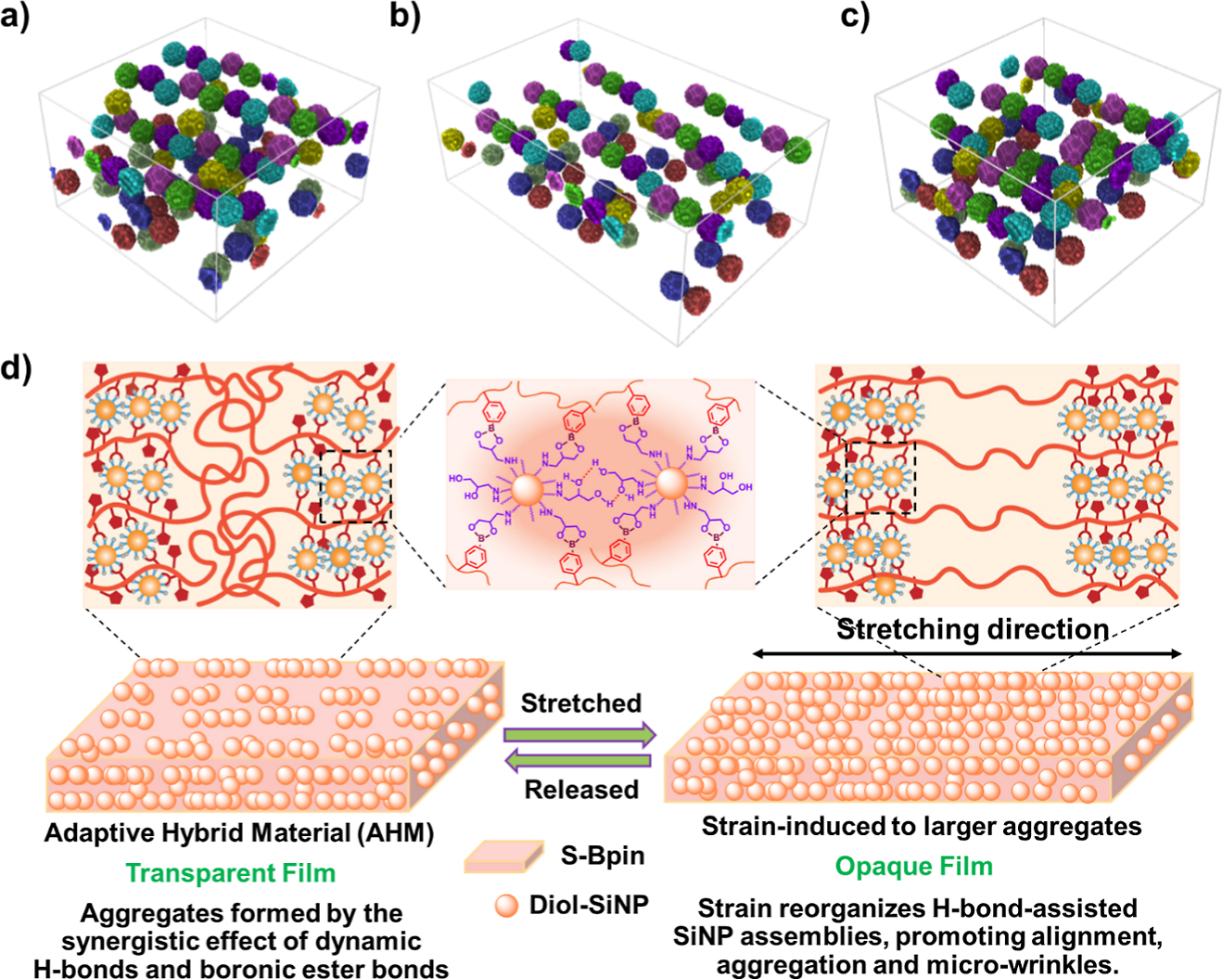
Proposed mechanism of reversible transparency. Reversible behavior of AHM obtained from coarse-grained (CG) MD simulations: (a) initial structure of NPs without strain, (b) elongated string-like clustered NPs after strain is applied, and (c) fully reversible structure [similar to (a)] once strain is released. While all the NPs are the same, for clarity, the NPs are colored differently, and also, the polymer matrix is not shown. Once strain is released, the AHM system comes back to the initial position, confirming reversibility. (d) A schematic illustration depicting nanoparticle orientation in a borylated styrene domain and showing a string-like nanoparticle orientation in unstrained (transparent) state. Under strain, elongation of the soft midblock drives nanoparticle alignment, larger aggregation (through H-bond reorganization), and oriented microwrinkle-assisted light scattering, producing the opaque state.

### Mechanism of Reversible and Tunable Mechano-Optic Responses

The combined structural, surface imaging, rheological, and MD results support a coupled mechanism for the reversible mechano-optical response of the AHM films. In the undeformed state, the films are transparent because the SiNP-rich domains are loosely organized and the refractive-index heterogeneity remains below the level needed for strong visible-light scattering. Diol-SiNPs are partially associated into stringlike domains through H-bonding among surface hydroxyl groups and are anchored to borylated styrene-rich domains through boronic ester linkages. At room temperature, these boronic ester linkages are not expected to undergo appreciable exchange during stretching; instead, they act as stable interfacial connections that transfer stress between the polymer matrix and nanoparticles. The combination of covalent anchoring and noncovalent association stabilizes the hybrid network while preserving the optical clarity.

Upon stretching, the soft poly­(ethylene-*co*-butylene) midblock elongates more readily than the stiffer borylated polystyrene-rich domains. This modulus contrast transfers stress to SiNP-rich regions, driving nanoparticle rearrangement, closer packing, and alignment along the strain direction (Figure 6D). H-bonding interactions among surface hydroxyl groups likely assist this reversible rearrangement, although their strain-dependent evolution was not directly measured spectroscopically. SAXS shows enhanced low-q scattering and anisotropic 2D patterns under strain, consistent with the formation of larger, oriented SiNP-rich aggregates, while TEM confirms clustered and stringlike nanoparticle assemblies. SEM further reveals that stretching produces oriented surface microwrinkles in aggregated 5 wt % diol-SiNP AHMs. These wrinkles arise from a local modulus mismatch between stiff aggregated SiNP/borylated-styrene-rich regions and the softer elastomeric midblock. The microwrinkles introduce additional surface scattering and amplify the opacity generated by the internal nanoparticle aggregation. In the well-dispersed diol-SiNP control, a reduced local stiffness contrast suppresses oriented wrinkle formation and prevents strong strain-induced opacity. Thus, oriented nanoparticle aggregation is essential for both internal scattering and microwrinkle-assisted optical switching.

Upon strain release, entropic elasticity of the poly­(ethylene-*co*-butylene) midblock drives macroscopic recovery. At the same time, most likely reversible hydrogen bond-assisted nanoparticle associations relax, allowing SiNP-rich domains to return to a metastable state. As internal aggregates relax and strain-induced microwrinkles disappear or become optically insignificant, refractive-index heterogeneity decreases and transparency is restored. Thermal stimulation provides a second mode of dynamic response: at elevated temperature, boronic ester exchange becomes active, enabling network relaxation that returns the system to its initial equilibrium state, allows reprocessing, and enables permanent shape reprogramming. Therefore, the reversible mechano-optical response of AHM operates in two coupled regimes: room-temperature optical switching governed mainly by reversible nanoparticle reorganization and surface microwrinkling, and high-temperature network reconfiguration governed by thermally activated boronic ester/interfacial exchange. This mechanism explains how interfacial dynamic chemistry, nanoparticle organization, and mechanical heterogeneity can be combined to design robust, optically reconfigurable, and reusable smart materials.

## Conclusions

In summary, we have developed an AHM that integrates boronic ester-based covalent linkages and H-bonding interactions within a triblock copolymer–SiNP interface, enabling reversible mechano-optical functionality combined with mechanical robustness. The strain-induced reversible transparency-to-opacity transition arises from a delicate interplay between polymer chain elasticity, nanoparticle alignment and aggregation under deformation, and surface microwrinkling. Upon stretching, SiNP-rich domains align and aggregate within the polymer matrix, while local modulus mismatch between stiff aggregated SiNP/borylated-styrene-rich regions and the softer poly­(ethylene-*co*-butylene) midblock promotes surface microwrinkling. These internal oriented aggregates and surface microwrinkles act as effective light-scattering centers under deformation. At room temperature, the mechano-optical response is governed primarily by physical reorganization of diol-SiNP assemblies and hydrogen-bond-assisted nanoparticle association, while boronic ester linkages serve as stable interfacial anchors for stress transfer. At elevated temperature, boronic ester exchange becomes active, enabling network relaxation, reprocessing, and permanent shape reprogramming. The optimized AHMs exhibit high tensile strength (∼39 MPa), toughness (∼83 MJ m^–3^), broad thermomechanical stability, and good cyclic mechano-optical stability under the tested conditions. Notably, the strained AHM film maintains its optical opaqueness for extended periods and even after prolonged high-temperature exposure. The films retain reversible optical switching after thermal reprocessing, demonstrating that the dynamic polymer–nanoparticle interface preserves both mechanical integrity and optical function. Overall, this work establishes a design strategy for mechanically robust, reprocessable, and optically adaptive soft materials by dual dynamic interfacial chemistry with controlled nanoparticle self-assembly and strain-induced surface instability. These findings provide a platform for adaptive optics, smart windows, soft robotics, sensing, reconfigurable coatings, and circular smart-material technologies.

## Methods

### Synthesis of Diol-SiNPs

At first, amino-functionalized SiNPs were synthesized from colloidal SiNPs. Colloidal SiNPs (20 g, 30% in MIBK), 3-aminopropyl­(dimethyl)­ethoxysilane (2 mL), and anhydrous THF (50 mL) were placed in a round-bottom flask. The solution was purged with argon for 30 min and heated under continuous stirring at 75 °C for overnight. Then, *n*-octyldimethylmethoxysilane (2 mL) was added to the reaction mixture and the reaction was continued overnight. After the reaction, the solution was cooled to room temperature and poured into 400 mL of hexane, and amino-functionalized SiNPs were recovered by centrifugation at 5000 rpm for 10 min. The dispersion–precipitation process was repeated another two times.

The amino-functionalized SiNPs were dispersed in anhydrous THF (30 mL) and purged with argon for 20 min. Then, glycidol (0.40 g) was added to the reaction mixture and stirred at 65 °C for 5.5 h. The gel-like reaction mixture was cooled down to room temperature and diluted with 10 mL of THF and 20 mL of hexane. The diol-SiNPs were recovered by centrifugation and stored under THF (10 mL) in a freezer. The product formation was confirmed by the significant weight gain observed in each step in TGA.

### Synthesis of AHM

AHM was prepared from S-Bpin and diol-SiNPs by following our previous report.^62^ S-Bpin (1.0 g) was dissolved in anhydrous THF (12 mL) in an oven-dried vial equipped with a stir bar. The solution was filtered using a 0.45 μm pore size filter to remove undissolved artifacts. A certain amount of diol-SiNP gel was dispersed in THF (2 mL) and added into the S-Bpin solution with continuous stirring. After 1 h of stirring at room temperature, solvents were dried under vacuum to give AHM as a rigid solid (the resulting mixture can be processed into films by solution casting as well). The AHM product was further dried at 120 °C under vacuum overnight to remove residual solvent. This partially cured AHM was hot-pressed at 215 °C for 30 min to 1 h with constant pressure (500 psi) to make the fully cured AHM film after slow cooling.

## Supplementary Material



## Notes

Materials, characterization methods, tensile analysis, DMA, rheology measurements, TEM, SEM, X-ray scattering (WAXS and SAXS), shape-memory property measurement, synthesis of diol-SiNPs and AHM, TGA and TEM plots of diol-SiNPs, control samples to show transparency changes, transmittance spectra of various controlled samples, multiple cycles stability, TEM images of 5 wt % diol-SiNP AHM, synthesis of well dispersed AHM with 10 wt % diol-SiNPs, optical microscopic images, SEM of the 5 wt % diol–SiNP AHM, AHMs’ thermal and mechanical properties, average tensile stress and strain at break of different diol-SiNP-loaded AHMs, cyclic tensile test under strain of 100% of 5 wt % diol-SiNP AHM, relaxation process and activation energy calculation, stress relaxation of 10 wt %, and 20 wt % AHM, metastable state and shape-memory behavior, and MD simulations (PDF)
