# Supplementary material for: Dynamic Interfacial Design in Adaptive Hybrid Materials Enables Reversible and Tunable Mechano-Optic Smart Responses
Source: ACS Nano. 2026 Jun 25;20(26):18644–57. doi: 10.1021/acsnano.5c22664 (PMC13348172; doi:10.1021/acsnano.5c22664)
Supplement: Supplementary file 1 [file nn5c22664_si_001.pdf]

Supporting Information

Dynamic Interfacial Design in Adaptive Hybrid Materials Enables Reversible and Tunable Mechano-Optic Smart Responses

Md Anisur Rahman,[1] Vera Bocharova,[1] Sungjin Kim,[1] Jihye Choi,[1,4] Bingrui Li,[1, 4] Sirui Ge,[2] Monojoy Goswami,[1] Xi Chelsea Chen,[1] Catalin Gainaru,[1] Alexei P. Sokolov,[1, 3] Tomonori Saito[1, 4]*

[1]Chemical Sciences Division, Oak Ridge National Laboratory, Oak Ridge, Tennessee 37831, United States

[2]Department of Materials Science and Engineering, University of Tennessee, Knoxville, Tennessee 37996, United States

[3]Department of Chemistry, University of Tennessee, Knoxville, Tennessee 37996, United States

[4]The Bredesen Center for Interdisciplinary Research and Graduate Education, University of Tennessee, Knoxville, Tennessee 37996, United States

[*]Corresponding author: Md Anisur Rahman. [*]Email: rahmana1@ornl.gov

The PDF file includes:

Supplementary Text

Figures S1-S15

Tables S1-S2

Supplementary Text

Materials. Polystyrene-*b*-poly(ethylene-*co*-butylene)-*b*-polystyrene (SEBS) (M_n = 118 kg/mol with Đ = 1.08; 18 mol % polystyrene repeating unit; 30 wt % polystyrene), 4,4′-di-tert-butyl bipyridine (dtbpy), chloro(1,5-cyclooctadiene)iridium(I) dimer ($[IrCl(COD)]_2$) and Glycidol were purchased from Sigma-Aldrich. Bis(pinacolato)diboron (B_2Pin_2) was purchased from oakwood chemicals. Silica nanoparticles (SiNPs) (MIBK-ST, 10-14 nm) mono-dispersed in methyl isobutyl ketone was donated by Nissan chemicals. The borylated SEBS (S-Bpin) was synthesized by following our previously reported method.[1] 3-aminopropyl(dimethyl)ethoxysilane, and n-octyldimethylmethoxysilane were purchased from Gelest and used directly without purification. Anhydrous tetrahydrofuran (THF) was purchased from Acros Organics and used as received. Methanol, dichloromethane (DCM), chloroform ($CHCl_3$), and dimethylformamide (DMF) were reagent grade and used without further purification.

Characterization Methods.^{1}H NMR spectra were recorded with a Bruker instrument (400 MHz) and signals were measured relative to the residual deuterochloroform ($CDCl_3$) signals for ^{1}H at 7.26 ppm. Infrared (IR) spectra were recorded on a Cary 600 Series FT-IR Spectrometer (Agilent Technologies). Differential scanning calorimetry (DSC) was performed on a DSC 250 from TA Instruments. Tzero pans were filled with 5-10 mg sample and were tested on a heat-cool-heat cycle at a ramp rate of 10 °C min^{-1} from -90 to 300 °C. Thermogravimetric analysis (TGA) of the functionalized SiNPs and composite samples were performed on TGA Q50, TA Instrument. Approximately 10 mg of each completely dry sample was weighted out to test TGA. The samples were heated up to 800 °C from room temperature at a heating rate of 20 °C min^{-1} under a dry nitrogen atmosphere (flow rate: 40 mL min^{-1}).

Tensile Analysis. Tensile stress and strain of polymer composite samples was measured using an Instron 3343 universal testing system equipped with 1 KN sensor following the ASTM D1708 standard. A punch was used to cut the dog-bone shape films with a width of 5 mm, a length of 22 mm and average thickness of 0.5 mm, which were tested at room temperature. Samples were elongated at the rate of 1 mm s^{-1} till break. Toughness was calculated from the area under the stress−strain curve. The mechanical properties were reported from an average of at least three specimens for each sample.

Dynamic Mechanical Analysis (DMA). Dynamic mechanical properties were carried out by TA Instruments DMA 850 using a tension clamp. Samples were prepared by hot pressing at 215 °C, and films were cut into rectangular shapes. Samples were tested in a temperature range from -120 °C to 300 °C at a rate of 3 °C min^{-1} with a frequency of 1 Hz and 15 μm amplitude.

Rheology Measurements. Rheological measurements were performed using an ARES G2 rheometer (TA Instruments) equipped with an 8 mm parallel plate geometry. Stress relaxation experiments were conducted at temperatures ranging from 230 °C to 260 °C under a constant strain of 1%. The relaxation modulus, G(t), was recorded as a function of time at each temperature. Prior to measurement, samples were thermally equilibrated for 5 minutes to ensure temperature stability. Master curves of the real and imaginary components of the complex shear modulus for the 5 wt % diol–SiNP AHM were constructed using 240 °C as the reference temperature. The corresponding master curve of the shear tan δ reveals the presence of a distinct relaxation process.

Transmission Electron Microscopy (TEM). For TEM analysis, thin sections approximately 100 nm thick were prepared by cryomicrotoming a piece of the bulk film at −80 °C using a Leica EM FC7 cryomicrotome. The sections were transferred onto lacey carbon-supported copper grids (Electron Microscopy Sciences) without staining. The dilute suspended solution of diol-SiNPs in THF was dropped into a lacey grid to take TEM images of diol-SiNPs. TEM imaging was conducted using an aberration-corrected FEI Titan S 80–300 TEM/STEM operated at an acceleration voltage of 300 keV.

Scanning Electron Microscopy (SEM). The top side of a piece of film was first sputtered with 2 nm of gold using an in-house built magnetron sputtering system. The film was stretched to about 100% strain and fixed onto a flat aluminum SEM stage with double-sided adhesive carbon tape. To ensure that the sample did not relax, the two ends of the stretched film were double secured by applying several layers of Kapton tape, leaving only about a third of the stretched length in the middle for SEM imaging. Unstretched samples were simply fixed onto the SEM stage with double-sided adhesive carbon tape. The samples were examined with a Zeiss Merlin SEM, using an acceleration voltage of 3 kV.

X-ray Scattering (SAXS and WAXS). X-ray scattering measurements were collected on a Xeuss 3.0 (Xenocs, France) equipped with a D2+ MetalJet X-ray source (Ga Kα, 9.2 keV, λ = 1.3414 Å). AHM films were adhered to the solids sample holder, aligned perpendicular to the direction of the incident beam (transmission mode), measured for 10 min and 20 min for wide angle and small angle measurements, respectively. Prior to data collection, LaB6 used to calibrate detector position for WAXS while silver behenate were used to calibrate detector position for SAXS. Two-dimensional scattering patterns were collected using an Eiger 2R 4M hybrid photon-counting detector (Dectris, Switzerland) with a pixel size of 75 × 75 μm². Azimuthal averaging of the 2D WAXS images was performed to obtain one-dimensional intensity profiles as a function of the scattering vector q, where $q = (4\pi \sin \theta)/\lambda$, following background subtraction and sample thickness corrections. The resulting 1D data were analyzed to extract information on nanoparticle morphology. In situ stretching experiments were carried out using a tensile frame integrated with the SAXS sample holder, enabling structural characterization under applied deformation.

Shape Memory Properties Measurement. Thermomechanical shape memory and shape reconfiguration were performed using DMA Q850 equipped with a tension clamp. Rectangular specimen (15 mm × 5 mm × 0.1 mm) was prepared for the measurement. To characterize the shape memory, the film specimen was first equilibrated at 120 °C ($T > T_g$) for 5 min, then deformed by increasing the applied force to 0.3 N at a rate of 0.03 N min^{-1}. The stretched sample was subsequently cooled to 0 °C ($T < T_g$) at a rate of 10 °C min^{-1} under constant force. Once cooled, the applied force was removed at the same rate (0.03 N min-1) to fix the temporary shape. The sample was then reheated to 140 °C and held for 10 min to trigger the shape recovery to the original shape. Following this, a shape reconfiguration cycle was performed to redefine the permanent shape. The sample was further heated to 200 °C, then stretched by ramping the force to 0.02 N at 0.002 N min^{-1} and equilibrated for 5 min. The specimen was then cooled to 140 °C and the applied force was removed at 0.002 N min^{-1} to fix the newly programmed permanent shape.

$$\text{Shape fixity } (R_f = \varepsilon_u/\varepsilon_l \times 100\%)$$
$$\text{Shape recovery } (R_r = (\varepsilon_u - \varepsilon_r)/\varepsilon_l \times 100\%)$$

ε_l is the maximum strain under load before unloading, ε_u is the strain after cooling and unloading, and ε_r is the strain after recovery measured during the shape memory cycle. ε_{load} and ε_{unload} are the

strains before and after load removal, respectively. Shape fixity and shape recovery at 140 °C is 85.2%, and 56.1% respectively. Shape fixity at 200 °C is 88.4%.

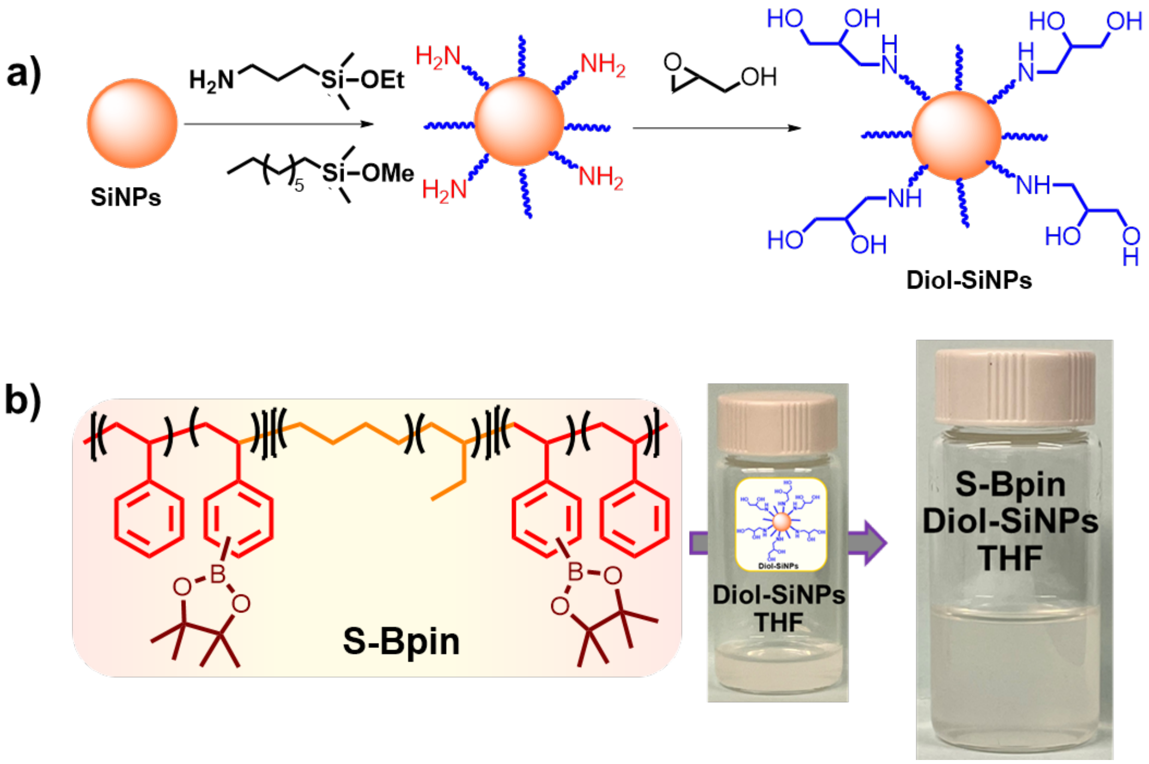

Figure S1. Synthesis of diol-SiNPs and AHM. **a)** Reaction scheme for the synthesis of diol-SiNPs. Amino functionalized SiNPs were first synthesized from neat SiNPs. Second step, diol-SiNPs were prepared from amine-SiNPs and glysidol. **b)** Synthesis of diol-SiNPs AHM, where diol-SiNPs was dissolved in THF then mixed with S-Bpin in THF solution that formed well dispersed cloudy solution. Photo credit: Md Anisur Rahman, ORNL.

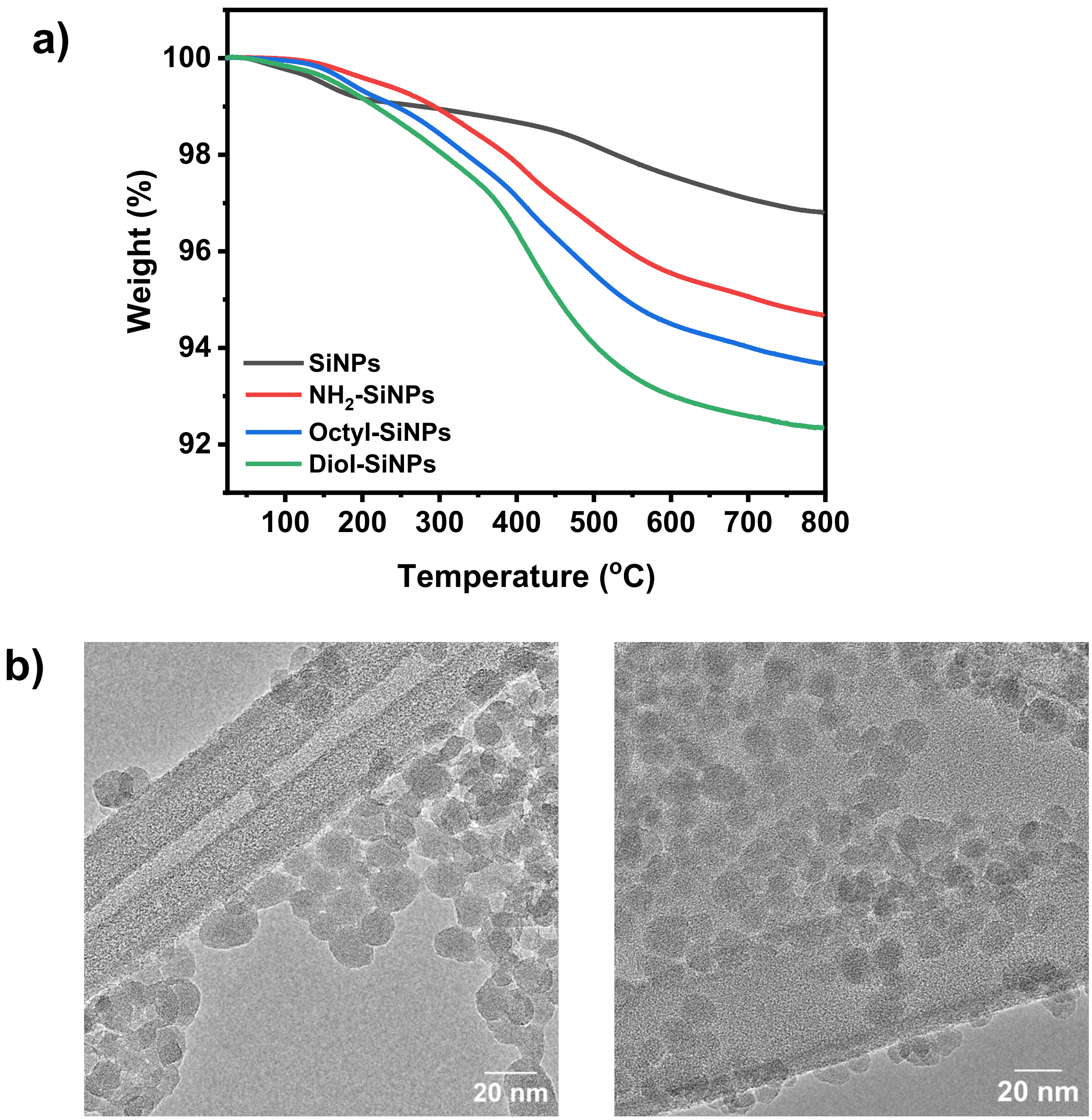

Figure S2. Thermogravimetric analysis (TGA) and TEM images of diol-SiNPs. **a)** TGA curves recorded from 25 to 800 °C at a heating rate of 10 °C min^{-1} demonstrate the stepwise functionalization process. Each modification stage shows a progressive increase in organic content, as evidenced by the incremental weight loss in the TGA profiles, confirming successful surface functionalization of the SiNPs. **b)** TEM images of diol-SiNPs were also showing the nanoparticles are interconnected/ aggregated.

Table S1. Table showing the photograpic images of different diol-SiNPs loaded AHMs and controlled samples at initial, stretched and released states. Photo credit: Md Anisur Rahman, ORNL.

Samples	Initial	Stretched	Released
5 wt% diol-SiNPs S-Bpin	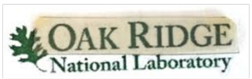	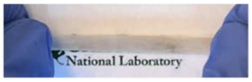	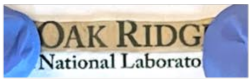
10 wt% diol-SiNPs S-Bpin	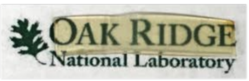	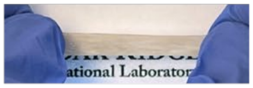	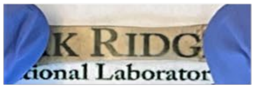
20 wt% diol-SiNPs S-Bpin	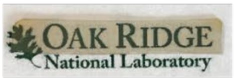	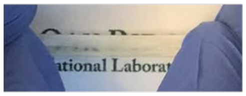	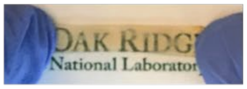
S-Bpin	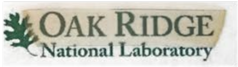	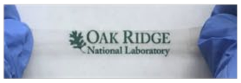	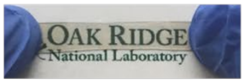
10 wt% SiNPs S-Bpin	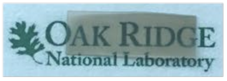	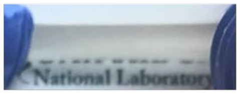	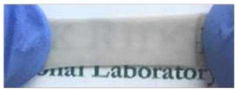
10 wt% SiNPs SEBS	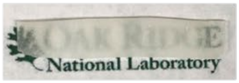	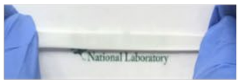	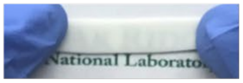
10 wt% diol-SiNPs SEBS	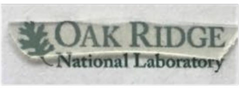	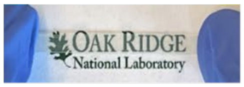	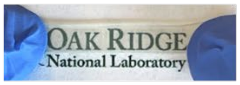

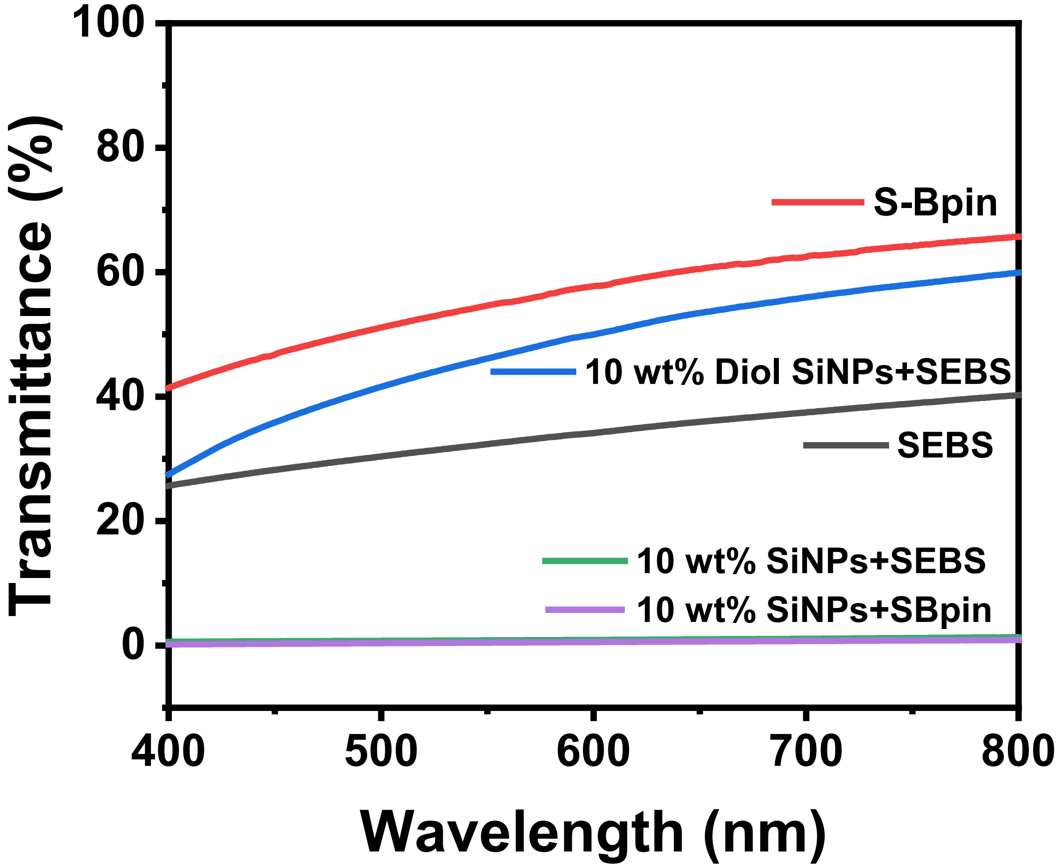

Figure S3. Transmittance spectra of various controlled samples at initial state (0% strain). We measured transmittence for S-Bpin, SEBS, loaded dynamic composites and controlled samples.

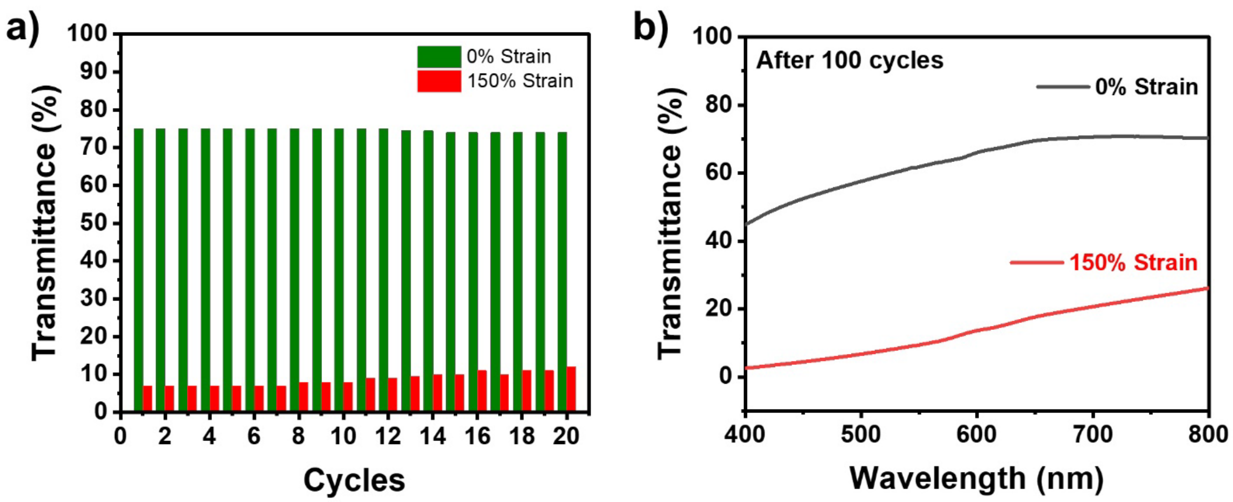

Figure S4. Multiple cycles stability. **a)** Transmittance of 5 wt% diol-SiNPs AHM film at 0% and 150% strain was measured at 700 nm wavelength for 20 cycles. **b)** Transmittance spectra of 5 wt% diol-SiNPs AHM film at 0% and 150% strain after 100 manual strain and released cycles.

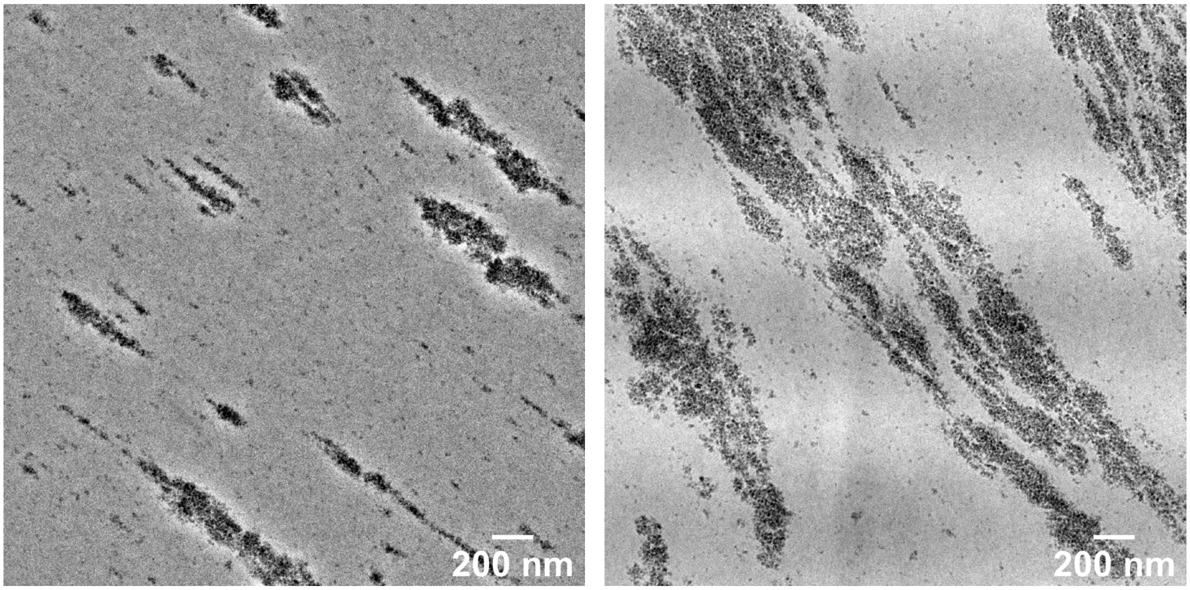

Figure S5. TEM images of 5 wt% diol-SiNPs AHM at different magnification. TEM images showed the nanoparticles form string like aggregated aligned structure.

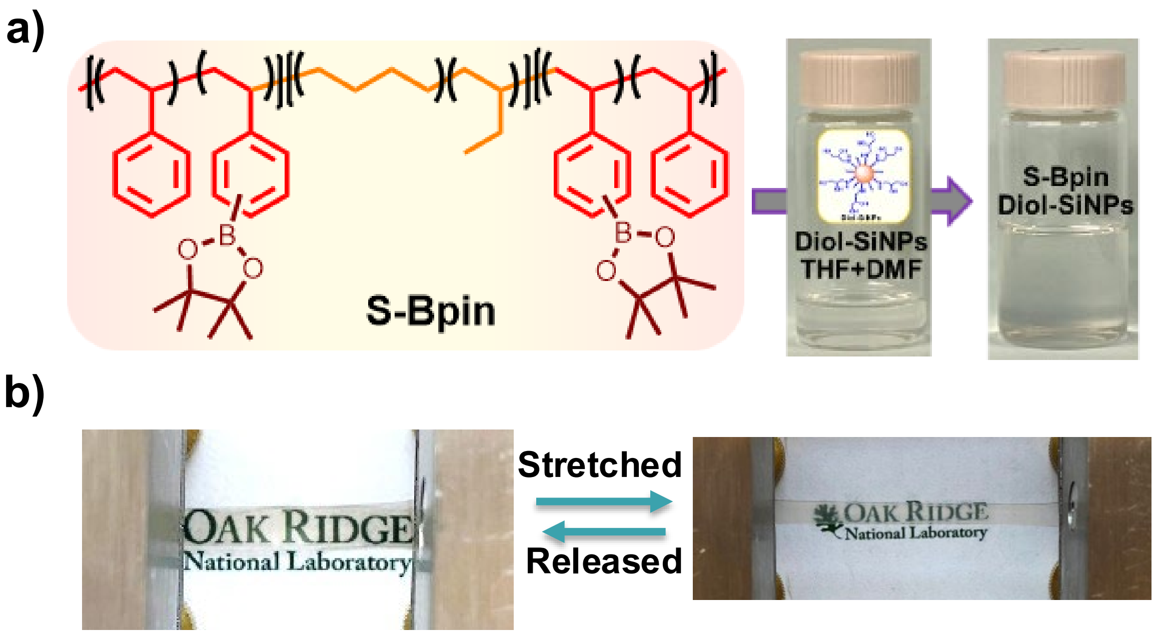

Figure S6. Synthesis of well dispersed AHM with 10 wt% diol-SiNPs. **a)** Schematic illustration of the reaction for preparing well-dispersed diol-SiNPs-containing AHM. Diol-SiNPs were first dissolved in a mixture of THF and DMF solvents, then combined with S-Bpin in THF to yield a clear and homogeneous solution, ensuring uniform nanoparticle dispersion throughout the composite. **b)** The image shows the strain-dependent transparency change. In this case, the film exhibit transparent even after 200% strain. Photo credit: Md Anisur Rahman, ORNL.

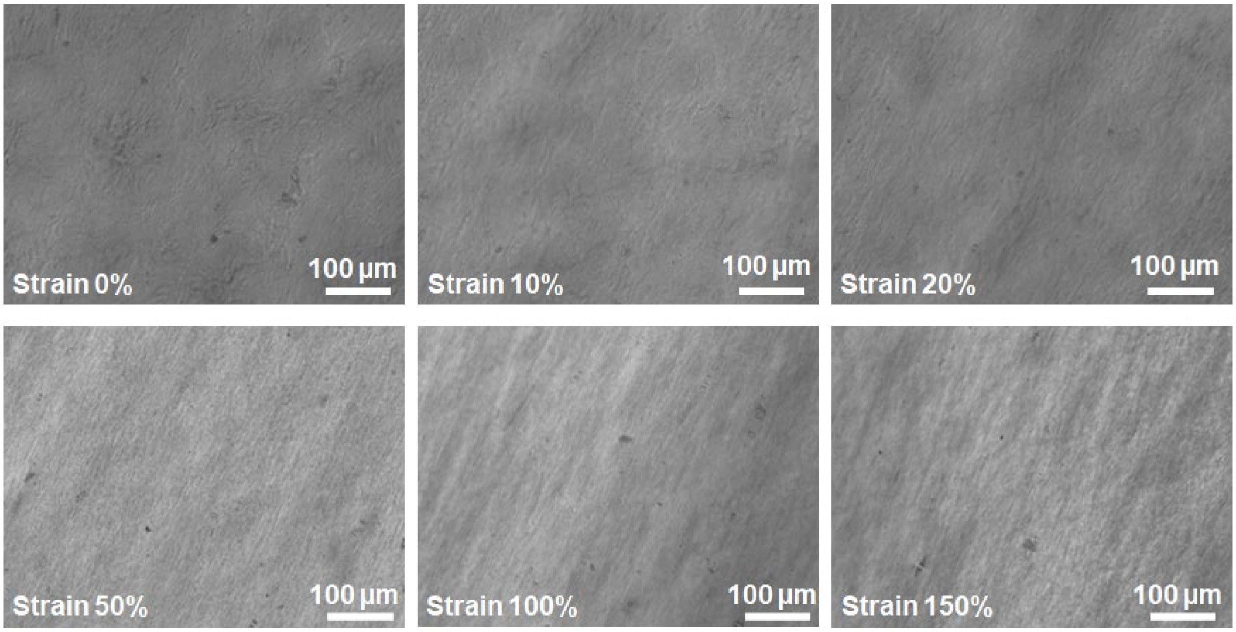

Figure S7. Optical microscopic images of 5 wt% diol-SiNPs loaded AHMs film at different strains.

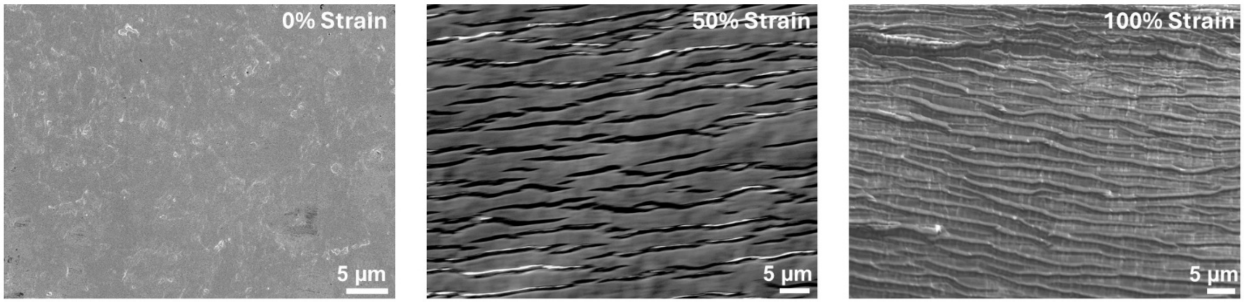

Figure S8. Scanning electron microscopy (SEM) of the 5 wt% diol–SiNP AHM. SEM image of 5 wt% AHM at 0% strain, 50% strain and 100% strain.

Table S2. AHMs' thermal and mechanical properties.

AHM samples	Glass transition temperature T_g (°C)	Tensile strength (MPa)	Tensile strain (%)	Toughness (MJ m^{-3})
**5 wt% diol-SiNPs**	-55, 186	37.0 ± 2.1	567 ± 38	83 ± 2.1
**10 wt% diol-SiNPs**	-55, 189	39.0 ± 2.5	462 ± 28	74 ± 1.4
**20 wt% diol-SiNPs**	-55, 192	33.5 ± 1.1	421 ± 23	70 ± 3.4

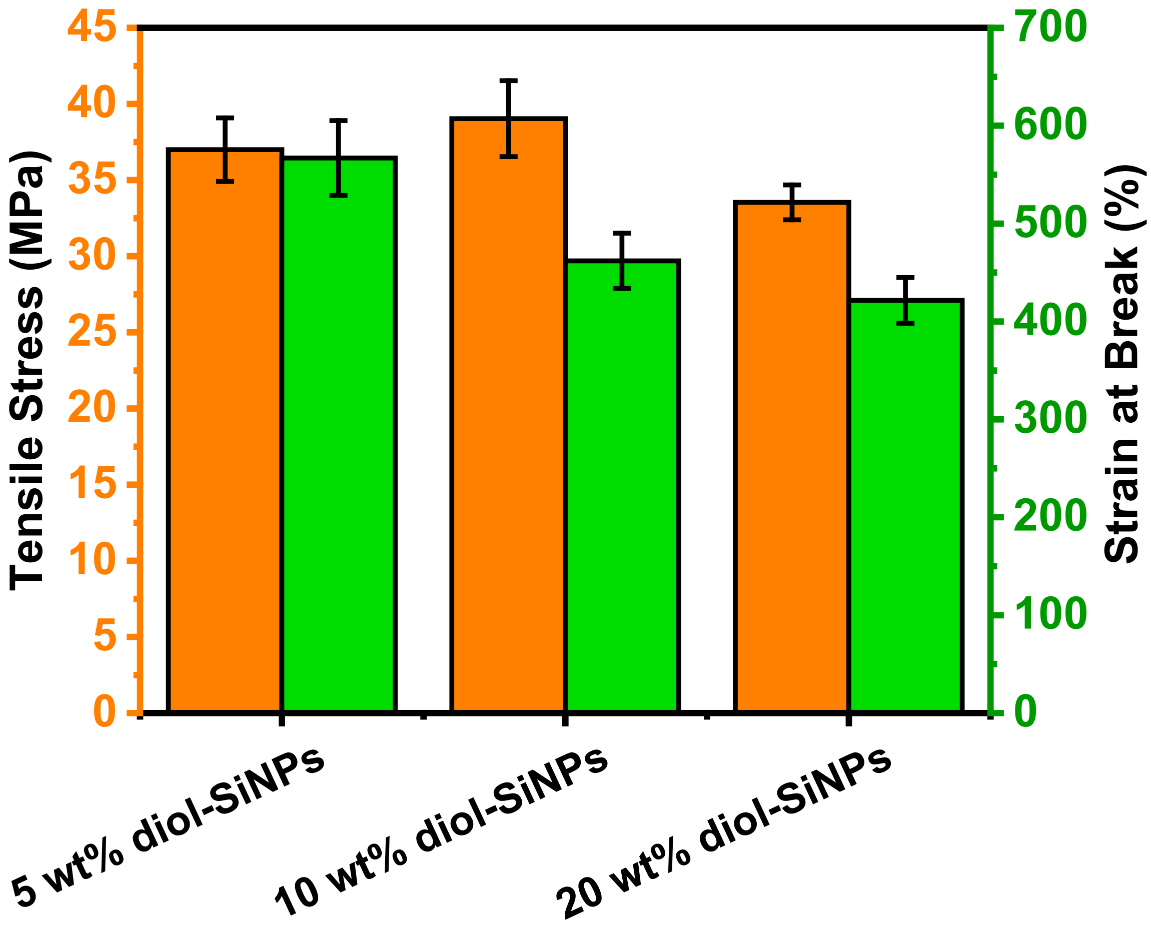

Figure S9. Average tensile stress and strain at break of different diol-SiNPs loaded AHMs.

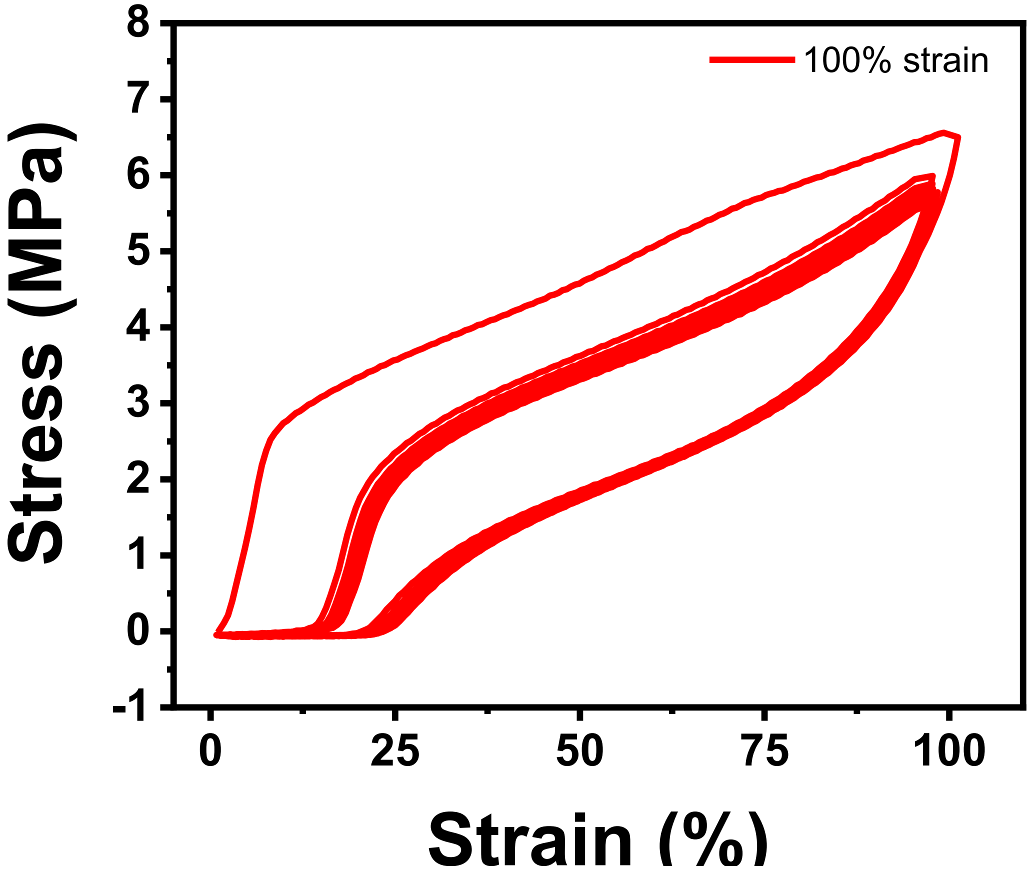

Figure S10. Cyclic tensile test under strain of 100% of 5 wt% diol-SiNPs AHM.

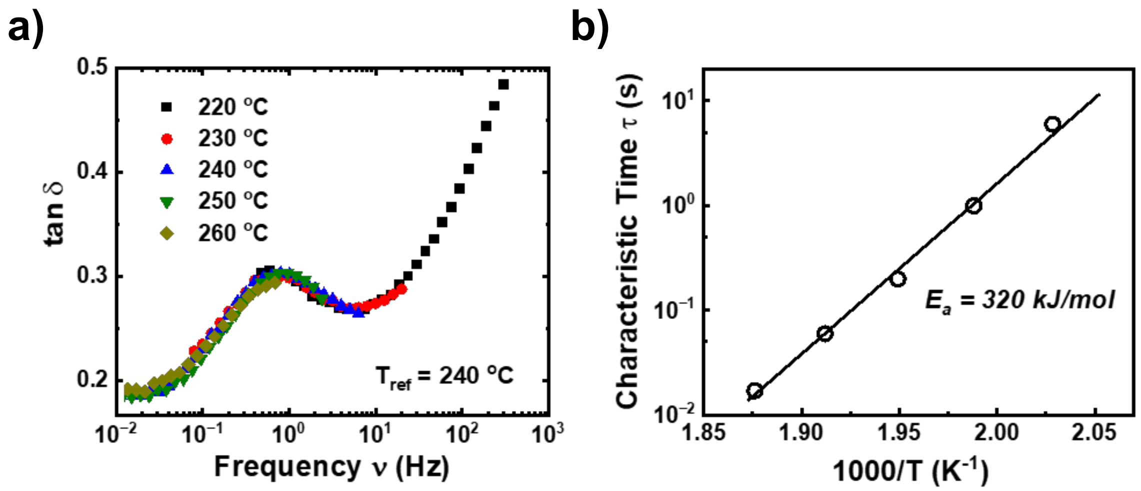

Figure S11. **a)** The corresponding master curve for shear tan delta reveals the presence of a relaxation process. **b)** The Arrhenius plot of the characteristic times for 5wt % diol-SiNP AHM, as obtained from the analysis of the shear tan delta spectra (open symbols). The solid line is an interpolation with an Arrhenius law, see text for details.

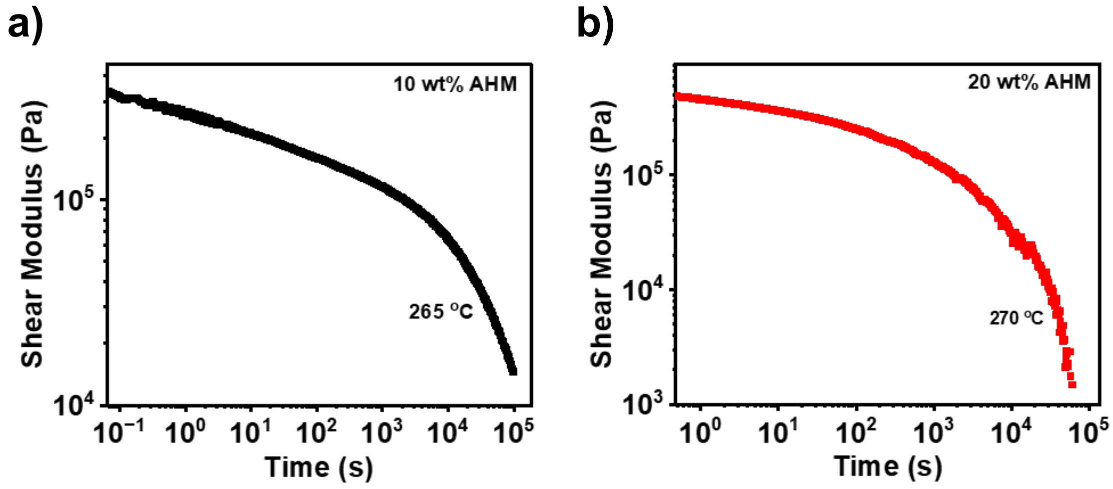

Figure S12. Stress relaxation of 10 wt% AHM (**a**) and 20 wt% AHM (**b**).

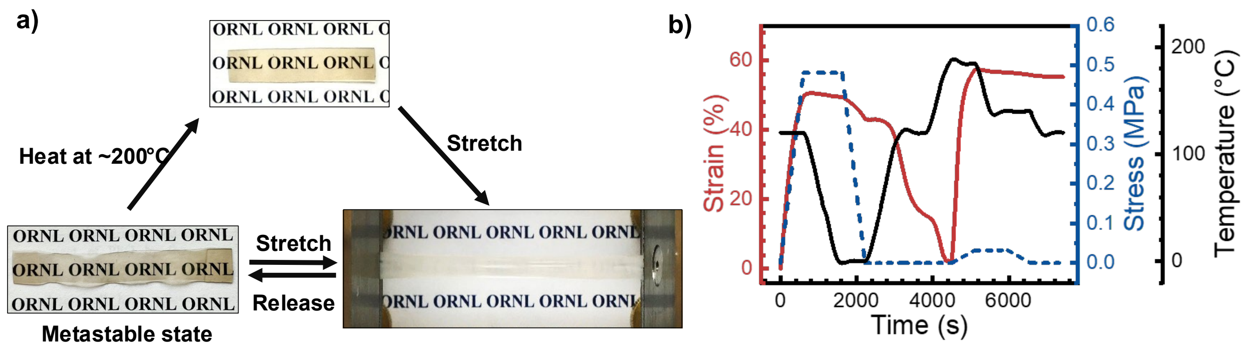

Figure S13. **a)** The AHM film enters a metastable state after being stretched and released. Heating to about 200 °C restores it to its original condition. **b)** Thermomechanical shape memory and shape reconfiguration of 5 wt% AHM film was measured by DMA. Photo credit: Md Anisur Rahman, ORNL.

Molecular Dynamics (MD) Simulations.

Coarse-grained (CG) molecular dynamics (MD) simulations were carried out following Kremer-Grest bead-spring polymer model in which polymer backbone is flexible with fixed rigid side beads representing styrene group as shown in Figure S14a. The beads are connected by Finite Extensible Nonlinear Elastic (FENE) bonds, and the inter-particle interactions are modeled using Lennard-Jones (LJ) potential with variable LJ interaction parameter, e. The NPs are modeled as spherical particles consisting of individual beads as shown in Figure S14b. The functionalization of the NPs is modeled by tuning (LJ) interaction parameters. The NP-side beads interactions are stronger representing strong interactions of rigid styrene beads with NP while the polymer backbone-NP interactions are weak. The NP box is then filled with polymers using Packmol[2] (Figure S14c). The NP box is then repeated in 3x3x1 times along x, y, and z direction, building a larger system as shown in Figure S10d. Thus, the total number of NP of larger box is 72. The larger simulation box is then compressed using LAMMPS 'change_box' command, followed by NPT simulation to obtain the required density of the system (Figure S14d). The final box size 80s×80s×50s as shown in Figure S14e, where s is the bead diameter. The interaction parameters, e, for the LJ is tuned to represent functionalization of the NPs by increasing the LJ interaction parameter between the side-beads of the chain and the NPs. The total number of particles in the system is 170,460 and the simulations are carried out at temperature, T* = 1.0 and P* = 1.0. The simulations used LJ units as described in LAMMPS (https://docs.lammps.org/units.html).

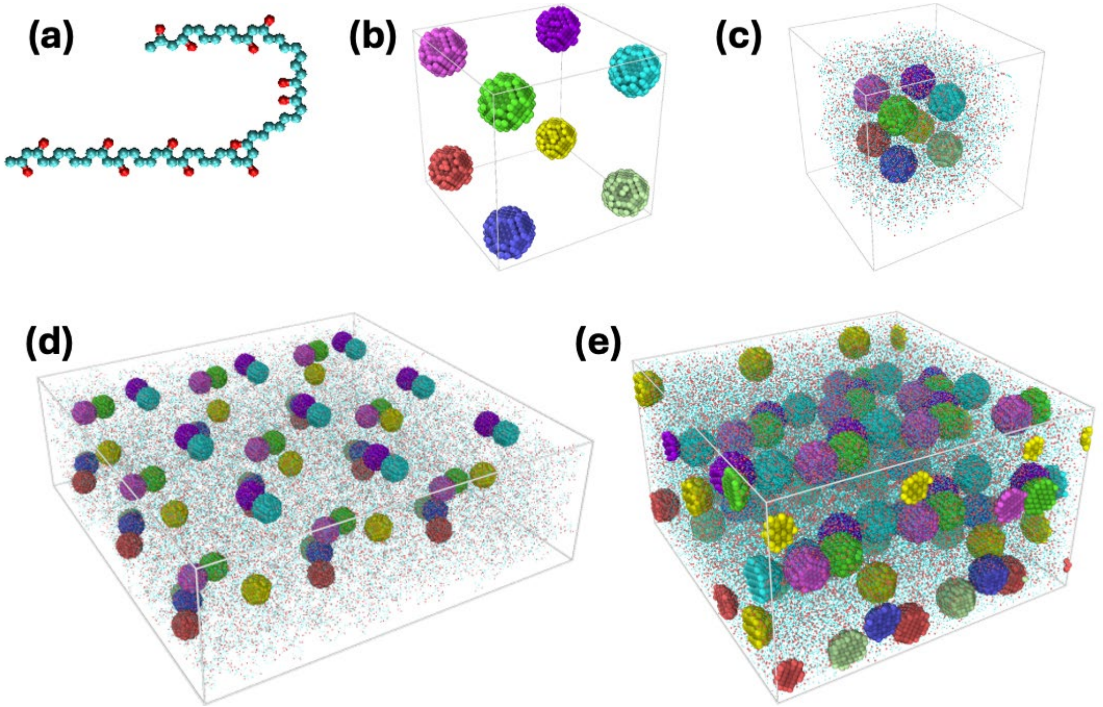

Figure S14. Initial system setup. **a)** the structure of the polymer, side-beads are rigid representing styrene of experimental material. **b)** Nanoparticles as developed initially. The colors of the SiNPs are shown different to distinguish the individual nanoparticles, property-wise they are the same representing functionalized SiNPs. **c)** Initial polymer filled SiNP system. For clarity the polymer beads are shown in small dots. **d)** Repeated box of (**c**) to develop larger system. **e)** Initial simulation system after minimization, the polymers are shown in small dots for clarity. The box of (**d**) is compressed to attain the required density.

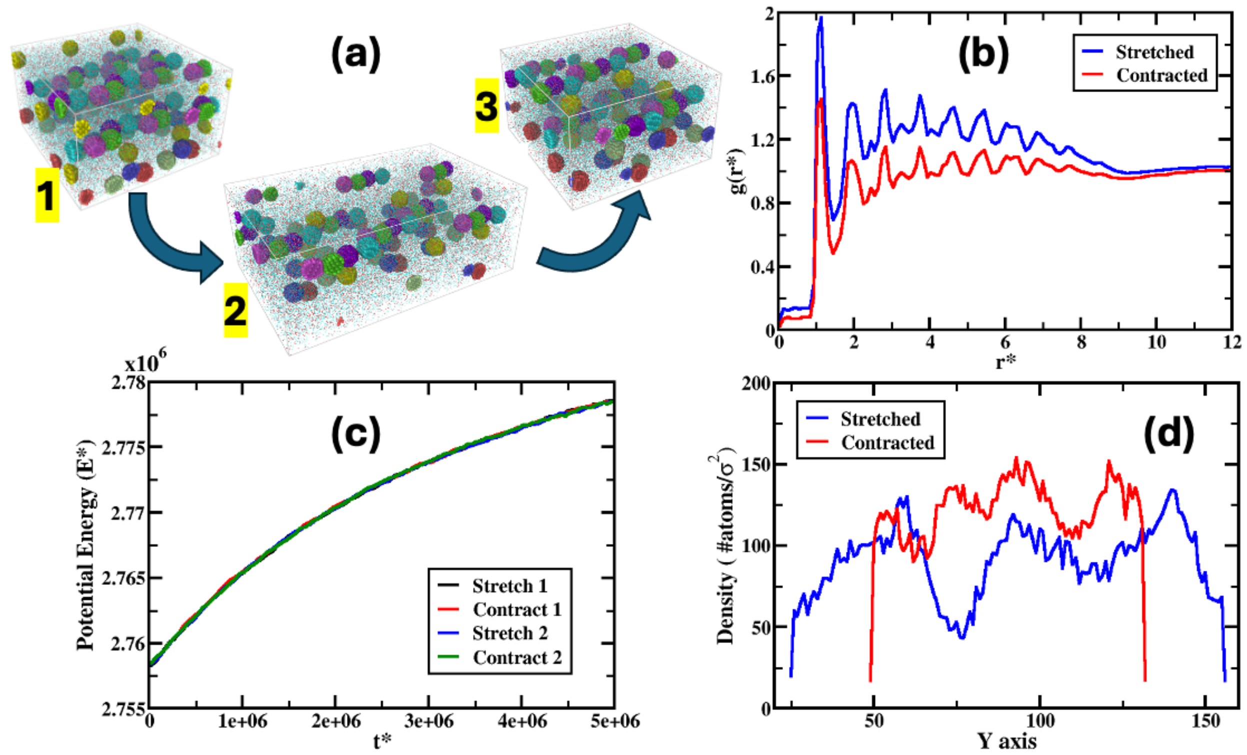

Figure S15. MD simulation results after long equilibration. **a**) Simulation snapshot of the polymer-NP system. After equilibration, the system looks like '1' before strain is applied. The applied strain along y-axis stretches the system as in '2' forming string-like structures of smaller NP clusters. In '3' the strain is released and it contracts back to its original state '1'. **b**) Radial distribution function (RDF) of side beads within 5s of the NPs. The blue curve shows when the strain is applied giving rise to higher peaks in layered structures. Red curve shows lower peaks, and hence weaker clustering when strain is released. **c**) Potential Energy (PE) per atom calculated when the strain is applied and released and repeated the strain application and release twice. PE was calculated by "compute pe/atom" command in LAMMPS that calculates PE of an atom due to its pair interactions and bond interactions with all other atoms. No change is PE represents reversible nature of these materials. **d**) Density distribution of the side-beads 5s away from the NPs. The peaks represent clustered NP positions. The broad peaks in stretched condition (strain application) represents string-like small clusters.

References

(1) Rahman, M. A.; Bowland, C.; Ge, S.; Acharya, S. R.; Kim, S.; Cooper, V. R.; Chen, X. C.; Irle, S.; Sokolov, A. P.; Savara, A.; et al. Design of tough adhesive from commodity thermoplastics through dynamic crosslinking. *Science Advances* **2021**, *7* (42), eabk2451.
(2) Martínez, L.; Andrade, R.; Birgin, E. G.; Martínez, J. M. PACKMOL: A package for building initial configurations for molecular dynamics simulations. *Journal of Computational Chemistry* **2009**, *30*, 2157-2164.
